# Terpene-Containing Analogues of Glitazars as Potential Therapeutic Agents for Metabolic Syndrome

**DOI:** 10.3390/cimb45030144

**Published:** 2023-03-08

**Authors:** Mikhail E. Blokhin, Sergey O. Kuranov, Mikhail V. Khvostov, Vladislav V. Fomenko, Olga A. Luzina, Natalia A. Zhukova, Cham Elhajjar, Tatiana G. Tolstikova, Nariman F. Salakhutdinov

**Affiliations:** N.N. Vorozhtsov Novosibirsk Institute of Organic Chemistry, 630090 Novosibirsk, Russia

**Keywords:** metabolic syndrome, glitazars, terpenic derivatives, isopimaric acid, OGTT, ITT

## Abstract

Metabolic syndrome is a complex of abnormalities involving impaired glucose and lipid metabolism, which needs effective pharmacotherapy. One way to reduce lipid and glucose levels associated with this pathology is the simultaneous activation of nuclear PPAR-alpha and gamma. For this purpose, we synthesized a number of potential agonists based on the pharmacophore fragment of glitazars with the inclusion of mono- or diterpenic moiety in the molecular structure. The study of their pharmacological activity in mice with obesity and type 2 diabetes mellitus (C57Bl/6^Ay^) revealed one substance that was capable of reducing the triglyceride levels in the liver and adipose tissue of mice by enhancing their catabolism and expressing a hypoglycemic effect connected with the sensitization of mice tissue to insulin. It has also been shown to have no toxic effects on the liver.

## 1. Introduction

Metabolic syndrome (MS) is a complex of pathological changes involving high blood sugar and abnormal cholesterol levels (decreased high-density lipoprotein and/or increased triglyceride levels, leading to hypertension and obesity and increasing the risk of cardiovascular disease, stroke, and heart attack [1]. Until recently, metabolic syndrome was predominantly suffered by the elderly (over 60 years of age), however, the picture has changed considerably over the last 20 years. Trends have shown that the problem is getting younger and more relevant to a younger population. In some countries, the proportion of adults suffering from these symptoms is as high as 25%. Over the past 20 years, the number of people worldwide with metabolic syndrome has increased by more than 100 million—a third [2].

There are two main disorders with this syndrome:

1. Type 2 diabetes mellitus. If lifestyle changes are not made and excess weight is not brought under control, insulin resistance can develop, which can cause high blood sugar levels, eventually leading to type 2 diabetes.
2. High cholesterol and high blood pressure contribute to the formation of plaques in the arteries. These plaques narrow the arteries’ openings, which can lead to a heart attack or stroke.

Recently, drugs from a new group that target both problems—glitazars—have been successfully developed [3]. Initially, these compounds were classified as glitazones, but a different mechanism of action, the activation of not only peroxisome proliferator-activated receptors gamma (PPAR-gamma) but also PPAR-alpha receptors, and changes in the structure features allowed them to be separated into the glitazar group. The drugs effectively influence the carbohydrate restoration and fat metabolism in patients with diabetes types 1 and 2, and have a favorable effect on the prevention and course of vascular complications [4].

*(S)*-2-Ethoxy-3-phenylpropanoic acid is considered to be a pharmacophore fragment common to glitazars. The main characteristic feature of this fragment is its binding to both PPAR-alpha and PPAR-gamma receptors, which allows compounds in this class of drugs to effectively regulate not only carbohydrate but also lipid metabolism.

**Figure 1:**
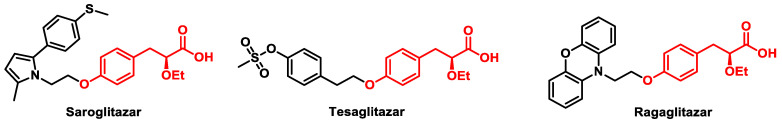
Glitazars–dual PPAR agonists.

Several of the dual PPAR agonists have shown promising results in animal studies and have subsequently been tested in clinical trials. The *(S)*-2-ethoxy-3-phenylpropanoic acid pharmacophore fragment is common to glitazars, but they all have a different variability. At present, only saroglitazar (Figure 1) has been approved for use, but only in India [5]. Ragaglitazar, tezaglitazar (Figure 1), and several others have failed in clinical trials due to the presence of various side-effects such as hepatotoxicity, cardiotoxicity, and gastrointestinal toxicity [6,7]. Recently, we proposed that the diverse side-effects of pharmacological agents are due to structural differences in the “tail” part of the glitazar molecule.

**Figure 2:**
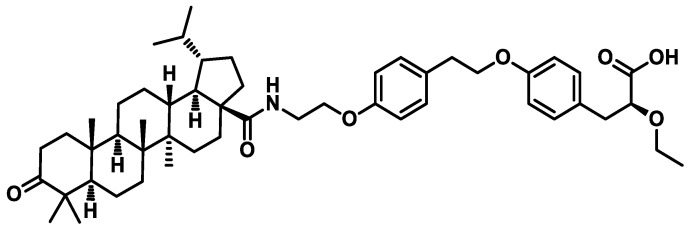
Dihydrobetulonic acid amide **BM-249**.

In a recent work, we showed that the use of triterpene acid fragments as the tail of the molecule imbues these compounds with hypoglycemic and hypolipidemic properties [8]. A synthesized compound **BM-249** (Figure 2), with a dihydrobetulonic acid fragment coupled to *(S)*-2-ethoxy-3-phenylpropanoic acid via an amide bond to an aminoethanol spacer, administered orally at a dose of 30 mg/kg for 5 weeks to mice on a high-fat/high-cholesterol diet (HF diet), showed an effect in reducing the blood glucose, total cholesterol (TC), and high-density lipoprotein (HDL) while having a relatively good safety profile.

The aim of this work was to synthesize analogues of compound **BM-249** containing other terpene fragments, namely, monoterpene and diterpene substituents, and to study their hypoglycemic and hypolipidemic properties.

## 2. Materials and Methods

### 2.1. Chemistry

The ^1^H and ^13^C NMR spectra for compounds were recorded on a Bruker AV-400 spectrometer (Bruker Corporation, Billerica, MA, USA) at 400.13 and 100.61 MHz, respectively in CDCl_3_ solution. The signals of the solvent were used as the reference (δH 7.27, δC 77.1 for CDCl_3_). Chemical shifts were given in ppm and the coupling constants (*J*) were given in hertz (Hz). The structure of the products was determined by means of 1H and 13C NMR spectra (Figures S1–S24). The mass spectra (15–500 m/z, 70 eV) were recorded on a DFS Thermo Scientific high-resolution mass spectrometer (Waltham, MA, USA). Merck silica gel (63–200 μm, Macherey-Nagel, Düren, Germany) was used for the column chromatography. Thin-layer chromatography was performed on TLC Silica gel 60F254 Merck (Darmstadt, Germany).

All reagents were used as described unless otherwise noted. Reagent-grade solvents were redistilled prior to use. Synthetic starting materials, reagents, and solvents were purchased from Sigma-Aldrich, Acros Organics (China) and Alfa Aesar (Germany).

All diterpenic acids were donated by colleagues from the Medicinal Chemistry Department. The reference compound tesaglitazar and ethyl *(S)*-2-ethoxy-3-(4-hydroxyphenyl) propanoate moiety were synthesized according to the methods in the literature [9]. The amine **5** containing ethyl *(S)*-2-ethoxy-3-(4-hydroxyphenyl) propanoate moiety was synthesized according to the procedure described earlier [8]. The obtained spectral data coincide with the literature data.

Synthesis of ethyl *(S)*-2-ethoxy-3-(4-(4-(4-(2-(monoterpene)aminoethoxy)phenethoxy)phenyl) propanoates, **6a,b**.

In a 50 mL flask, 4 mmol of *(S)*-ethyl 3-(4-(4-(2-(2-aminoethoxy)-phenethoxy) phenyl)-2-ethoxy propanoate **5**, 3.9 mmol of the corresponding monoterpene aldehyde in 10 mL of methylene chloride, and then 6.4 mmol NaBH(OAc)_3_ was added in portions. The reaction mixture was stirred at room temperature for 12 h. Then, the mixture was diluted with 15 mL of water, 4 mL of 1 N NaOH solution, and left to stir for 20 min. Next, the mixture was extracted, the organic layer was washed with saturated NaCl solution, and dried over magnesium sulfate. The purification was conducted by column chromatography (eluent: CHCl_3_:MeOH-100:1).

Ethyl (2S)-3-[4-(2-{4-[2-({[(1R,5S)-6,6-dimethylbicyclo [3.1.1]hept-2-en-2-yl]methyl} amino)ethoxy]phenyl}ethoxy)phenyl]-2-ethoxypropanoate, **6a**.

Yellow oil, 86% yield. ^1^H-NMR: 0.85 (3 H, s), 1.13–1.20 (3 H, m), 1.20–1.26 (3 H, m), 1.29 (3 H, s), 1.68 (1 H, br.s), 2.11 (2 H, d, J = 5.5), 2.18–2.35 (2 H, m), 2.39 (1 H, d.t., *J =* 8.6, 5.6), 2.92–3.00 (4 H, m), 3.03 (2 H, t, *J =* 7.1), 3.19 (2 H, m), 3.35 (1 H, d.q., *J =* 9.1, 7.0), 3.60 (1 H, d.q., *J =* 9.1, 7.0), 3.97 (1 H, dd, *J =* 7.3, 6.0), 4.04–4.21 (6 H, m), 5.38–5.42 (1 H, m), 6.79–6.85 (2 H, m), 6.85–6.89 (2 H, m), 7.15 (2 H, d, *J =* 8.6), 7.19 (2 H, d, *J =* 8.6). ^13^C-NMR: 14.2, 15.0, 21.0, 26.2, 31.2, 31.6, 34.9, 38.0, 38.4, 40.9, 44.3, 48.2, 54.5, 60.7, 66.1, 67.3, 68.8, 80.4, 114.2 (2C), 114.4 (2C), 117.6, 129.2, 129.9 (2C), 130.3 (2C), 146.3, 157.5, 172.5. Found: *m*/*z* 535.3298 [M]+. C_33_H_45_NO_5_. Calculated: M 535.3298.

Ethyl (2S)-3-{4-[2-(4-{2-[(3,7-dimethyloct-6-en-1-yl)-amino]-ethoxy}-phenyl)-ethoxy] phenyl}-2-ethoxypropanoate, **6b**.

Colorless oil, 75% yield. ^1^H-NMR: 0.83–0.98 (3 H, m), 1.17 (3 H, t, *J =* 7.1), 1.23 (3 H, t, *J =* 7.1), 1.28–1.44 (2 H, m), 1.45–1.55 (2 H, m), 1.67–1.75 (3 H, m), 1.76–1.8 (3 H, m), 1.86–2.11 (3 H, m), 2.62–2.79 (2 H, m), 2.91–2.98 (2 H, m), 2.98–3.07 (4 H, m), 3.35 (1 H, d.q., *J =* 9.0, 7.0), 3.60 (1 H, d.q., *J =* 9.1, 7.0), 3.96 (1 H, t, *J =* 6.6), 4.04–4.26 (6 H, m), 5.06–5.14 (1 H, m), 6.81 (4 H, m), 7.15 (4 H, m). ^13^C-NMR: 14.2, 15.0, 17.6, 19.6, 25.4, 25.7, 30.6, 34.9, 37.0, 37.1, 38.4, 47.7, 48.8, 60.7, 66.1, 67.1, 68.8, 80.4, 114.3 (2C), 114.5 (2C), 124.7, 129.2, 129.9 (2C), 130.3 (2C), 130.4, 131.2, 157.4, 157.5, 172.5. Found: *m*/*z* 539.3611 [M]+. C_33_H_49_NO_5_. Calculated: M 539.3610.

Synthesis of amides **8a–d**.

In a 50 mL round bottom flask with 20 mL of N,N-dimethylformamide (DMF), the corresponding diterpene acid (2.5 mmol) and amine 5, 1.1 g (2.75 mmol), were dissolved, then 0.72 g (1.9 mmol) 2-(1H-benzotriazol-1-yl)-1,1,3,3-tetramethyluronium hexafluorophosphate (HBTU) was added. Next, 0.54 g (4.2 mmol) of diisopropylethylamine (DIPEA) was added on cooling in an ice bath. The reaction was carried out in an inert atmosphere under stirring at room temperature for 5 h. The reaction was treated by dilution with water, followed by acidification with 10% hydrochloric acid to pH~2–3 and extraction with EtOAc. The organic phase was washed with saturated NaHCO_3_ solution and dried over MgSO_4_. The purification was conducted by column chromatography on silica gel using the system hexane:EtOAc-3:1.

Ethyl (2S)-3-(4-{2-[4-(2-{[(1R,4aR,7S)-7-ethenyl-1,4a,7-trimethyl-1,2,3,4,4a,4b,5,6,7, 8,10,10a-dodecahydrophenanthren-1-yl]formamido}ethoxy)phenyl]ethoxy}phenyl)-2-ethoxypropanoate, **8a**.

Yellow oil, 79% yield. ^1^H-NMR: 0.78–0.88 (8 H, m), 1.15–1.18 (8 H, m), 1.20–1.25 (3 H, m), 1.25–1.99 (12 H, m), 2.87–2.93 (2 H, m), 3.05 (2 H, m), 3.25–3.36 (1 H, m), 3.51–3.71 (3 H, m), 3.98 (3 H, m), 4.03–4.16 (4 H, m), 4.83 (2 H, m), 5.23 (1 H, m), 5.81 (1 H, m), 6.19–6.28 (1 H, m), 6.79 (4 H, dd, *J =* 14.4, 8.7), 7.07–7.19 (4 H, m). ^13^C-NMR: 14.2, 15.0, 15.3, 17.2, 17.9, 19.9, 21.4, 24.7, 25.1, 34.8, 36.0, 36.7, 38.7, 39.1, 44.9, 45.6, 44.9, 46.0, 46.3, 51.9, 60.7, 66.1, 66.8, 68.7, 80.3, 109.2, 114.2 (2C), 114.5 (2C), 120.6, 129.2, 130.0 (2C), 130.3 (2C), 130.9, 135.5, 150.3, 157.1, 157.4, 172.5, 178.9. Found: *m*/*z* 685.4342 [M]+. C_43_H_59_NO_6_. Calculated: M 685.4343.

Ethyl (2S)-3-(4-{2-[4-(2-{[(1R,4aS,10aR)-1,4a-dimethyl-7-(propan-2-yl)-1,2,3,4,4a,9, 10,10a-octahydrophenanthren-1-yl]formamido}ethoxy)phenyl]ethoxy}phenyl)-2-ethoxypropanoate, **8b**.

Orange oil, 76% yield. ^1^H-NMR: 1.13 (3 H, t, *J =* 7.0), 1.16–1.25 (12 H, m), 1.42 (3 H, s), 1.41–1.75 (7 H, m), 2.12–2.21 (1 H, m), 2.22–2.31 (1 H, m), 2.77 (3 H, s), 2.89–2.94 (2 H, m), 3.10 (2 H, m), 3.26–3.36 (1 H, m), 3.51–3.72 (3 H, m), 3.90–4.03 (3 H, m), 4.04–4.17 (4 H, m), 6.22–6.35 (1 H, m), 6.80 (5 H, dd, *J =* 14.2, 8.7), 6.93–6.99 (1 H, m), 7.08–7.21 (5 H, m). ^13^C-NMR: 14.1, 15.0, 16.3, 18.6, 21.0, 23.9 (2C), 25.1, 29.8, 33.3, 34.8, 37.0, 37.8, 38.4, 39.2, 45.4, 47.2, 60.7, 66.1, 66.7, 68.7, 80.2, 114.1 (2C), 114.4 (2C), 123.7, 124.0, 126.8, 129.1, 123.0 (2C), 130.3 (2C), 130.8, 134.5, 145.5, 146.8, 157.1, 157.4, 172.5, 178.5. Found: *m*/*z* 683.4186 [M]+. C_43_H_57_NO_6_. Calculated: M 683.4186.

Ethyl (2S)-3-(4-{2-[4-(2-{[(1R,4aR,10aR)-1,4a-dimethyl-7-(propan-2-yl)-1,2,3,4,4a,4b,5, 6,10,10a-decahydrophenanthren-1-yl]formamido}ethoxy)phenyl]ethoxy}phenyl)-2-ethoxypropanoate, **8c**.

Yellow oil, 71% yield. ^1^H-NMR: 0.75–0.8 (3 H, m), 0.97 (5 H, m), 1.17–1.26 (10 H, m), 1.42–1.58 (3 H, m), 1.65–1.98 (7 H, m), 2.10–2.22 (1 H, m), 2.88–2.94 (2 H, m), 3.00 (2 H, t, *J =* 7.1), 3.31 (1 H, dd, *J =* 9.1, 7.1), 3.52–3.69 (3 H, m), 3.93 (1 H, dd, *J =* 7.2, 6.1), 3.99 (2 H, t, *J =* 5.1), 4.05–4.12 (7 H, m), 5.23 (1 H, d, *J =* 4.8), 5.70 (1 H, s), 6.18 (1 H, br. s.), 6.80 (4 H, dd, *J =* 15.4, 8.6), 7.11 (2 H, d, *J =* 8.6), 7.17 (2 H, d, *J =* 8.5). ^13^C-NMR: 14.1, 14.2 (2C), 15.0, 16.9, 18.2, 20.8, 21.4, 22.4, 23.9, 25.2, 27.3, 29.7, 34.5, 34.8, 37.1, 37.8, 38.4, 39.2, 45.6, 46.3, 50.9, 60.4, 66.1, 66.8, 68.8, 80.4, 114.2 (2C), 114.6 (2C), 120.4, 122.3, 129.2, 130.0 (2C), 130.3 (2C), 130.9, 135.4, 145.1, 157.2, 157.5, 172.5, 178.5. Found: *m*/*z* 685.4342 [M]+. C_43_H_59_NO_6_. Calculated: M 685.4342.

Ethyl (2S)-3-(4-{2-[4-(2-{[(1R,4aR,5S)-5-[2-(furan-3-yl)ethyl]-1,4a-dimethyl-6-methyl idenedecahydronaphthalen-1-yl]formamido}ethoxy)phenyl]ethoxy}phenyl)2ethoxypropanoate, **8d**.

Yellow oil, 68% yield. ^1^H-NMR: 0.55 (3 H, s), 1.00 (1 H, td, *J =* 13.1, 3.7), 1.09–1.17 (6 H, m), 1.17–1.30 (5 H, m), 1.47–1.62 (2 H, m), 1.68 (3 H, m), 1.77–1.93 (5 H, m), 1.96–2.09 (2 H, m), 2.14–2.25 (1 H, m), 2.40 (1 H, dd, *J =* 8.3, 2.1), 2.46–2.56 (1 H, m), 2.88–2.95 (2 H, m), 3.00 (2 H, t, *J =* 7.1), 3.32 (1 H, dq, *J =* 9.1, 7.1), 3.51–3.67 (3 H, m), 3.91–4.04 (3 H, m), 4.04–4.19 (4 H, m), 4.50 (1 H, s), 4.82 (1 H, s), 6.06 (1 H, t, *J =* 5.4), 6.22 (1 H, d, *J =* 0.7), 6.72–6.86 (4 H, m), 7.09–7.21 (5 H, m), 7.31 (1 H, t, *J =* 1.5). ^13^C-NMR: 12.6, 14.1, 15.0, 20.0, 23.4, 24.1, 26.6, 30.0, 34.8, 38.2, 38.4, 38.7, 38.8, 39.2, 40.2, 44.0, 55.0, 56.4, 60.7, 66.1, 66.4, 68.7, 80.3, 106.5, 110.9, 114.2 (2C), 114.4 (2C), 125.3, 129.2, 130.0 (2C), 130.3 (2C), 130.7, 138.6, 142.6, 147.4, 157.0, 157.4, 172.5, 176.7. Found: *m*/*z* 699.4135 [M]+. C_43_H_57_NO_7_. Calculated: M 699.4134.

Hydrolysis of compounds **6a,b,8a–d**.

Hydrolysis of the ester group of the compounds obtained was performed according to the procedure described in our previous work [8].

{2-[4-(2-{4-[(2S)-2-carboxy-2-ethoxyethyl]phenoxy}ethyl)phenoxy]ethyl}({[(1R,5S)-6,6-dimethylbicyclo [3.1.1]hept-2-en-2-yl]methyl})azanium chloride, **7a**.

Colorless oil, 87% yield. ^1^H NMR: 0.83 (3 H, s), 1.05–1.21 (3 H, m), 1.22–1.43 (3 H, m), 1.98–2.13 (2 H, m), 2.19–2.37 (2 H, m), 2.39–2.50 (2 H, m), 2.91–3.04 (4 H, m), 3.26 (3 H, m), 3.52–3.68 (4 H, m), 3.96 (1 H, dd, *J =* 7.9, 4.4), 4.00–4.09 (2 H, m), 4.28 (2 H, br.s.), 5.78 (1 H, br. s.), 6.75 (2 H, d, *J =* 8.6), 6.86 (2 H, d, *J =* 8.5), 7.13 (4 H, dd, *J =* 13.0, 8.6). ^13^C-NMR: 13.8, 15.0, 18.8, 20.7, 21.0, 25.8, 29.6, 31.5, 31.5, 34.6, 34.8, 37.9, 38.0, 40.0, 43.6, 44.8, 51.7, 62.5, 62.9, 66.4, 68.6, 79.7, 114.2 (2C), 114.6 (2C), 127.4, 129.0, 129.9 (2C), 130.5 (2C), 131.3, 138.3, 156.1, 157.4, 175.8, 176.1. Found: *m*/*z* 507.2985 [M]^+^. C_31_H_41_NO_5_. Calculated: M 507.2985.

{2-[4-(2-{4-[(2S)-2-carboxy-2-ethoxyethyl]phenoxy}ethyl)phenoxy]ethyl}(3,7-dimethyloct-6-en-1-yl)azanium chloride, **7b**.

Colorless oil, 91% yield. ^1^H-NMR: 0.84–0.96 (3 H, m), 1.09–1.20 (3 H, m), 1.24–1.40 (3 H, m), 1.51 (1 H, d, *J =* 6.2), 1.59 (3 H, s), 1.64–1.75 (4 H, m), 1.80–2.07 (3 H, m), 2.81–3.00 (4 H, m), 3.07 (2 H, dtt, *J =* 17.4, 11.7, 11.7, 5.6, 5.6), 3.23 (2 H, m), 3.33–3.46 (1 H, m), 3.50–3.62 (1 H, m), 3.94–4.05 (3 H, m), 4.15–4.26 (2 H, m), 5.04 (1 H, t, *J =* 6.9), 6.71 (2 H, d, *J =* 8.6), 6.78–6.88 (2 H, m), 7.10 (4 H, dd, *J =* 18.9, 8.5). ^13^C-NMR: 15.0, 17.7, 19.0, 25.2, 25.7, 30.4, 32.6, 34.9, 36.5, 37.8, 46.2, 46.48, 63.0, 66.2, 68.6, 76.6, 77.3, 80.0, 114.2 (2C), 114.6 (2C), 124.1, 128.9 (2C), 130.0 (2C), 130.4, 131.4, 131.6, 156.1, 157.5, 175.5. Found: *m*/*z* 511.3298 [M]^+^. C_31_H_45_NO_5_. Calculated: M 511.3297.

(2S)-3-(4-{2-[4-(2-{[(1R,4aR,7S)-7-ethenyl-1,4a,7-trimethyl-1,2,3,4,4a,4b,5,6,7,8,10, 10a-dodecahydrophenanthren-1-yl]formamido}ethoxy)phenyl]ethoxy}phenyl)-2-ethoxy-propanoic acid, **9a**.

Yellow oil, 85% yield. ^1^H-NMR: 0.78–0.89 (6 H, m), 1.05–1.16 (5 H, m), 1.27–1.38 (2 H, m), 1.39–1.58 (7 H, m), 1.58–2.04 (9 H, m), 2.84–3.05 (4 H, m), 3.37 (1 H, dd, *J =* 8.8, 7.3), 3.52–3.66 (3 H, m), 3.93–4.01 (3 H, m), 4.07 (2 H, t, *J =* 7.0), 4.80–4.93 (2 H, m), 5.14–5.31 (1 H, m), 5.71–5.82 (1 H, m), 6.25 (1 H, t, *J =* 5.2), 6.80 (4 H, dd, *J =* 16.5, 8.4), 7.09–7.21 (4 H, m). ^13^C-NMR: 15.0, 15.3, 17.3, 17.5, 19.9, 21.4, 21.2, 24.7, 34.8, 36.0, 36.7, 38.4, 38.7, 39.2, 45.6, 46.0, 52.0, 66.1, 66.8, 68.7, 80.3, 109.2, 114.2 (2C), 114.5 (2C), 120.8, 120.9, 129.2, 1230.0 (2C), 130.3 (2C), 130.8, 135.5, 135.6, 150.2, 157.1, 157.5, 172.5, 178.9. Found: *m*/*z* 657.4029 [M]+. C_41_H_55_NO_6_. Calculated: M 657.4028.

(2S)-3-(4-{2-[4-(2-{[(1R,4aS,10aR)-1,4a-dimethyl-7-(propan-2-yl)-1,2,3,4,4a,9,10,10a-octahydrophenanthren-1-yl]formamido}ethoxy)phenyl]ethoxy}phenyl)-2-ethoxypropanoic acid, **9b**.

Yellow oil, 88% yield. ^1^H-NMR: 1.10–1.30 (15 H, m), 1.38–1.58 (3 H, m), 1.63–1.77 (4 H, m), 2.11 (1 H, d, *J =* 12.2), 2.28 (1 H, d, *J =* 12.1), 2.72–3.07 (7 H, m), 3.38 (1 H, dd, *J =* 8.1, 7.8), 3.51–3.70 (3 H, m), 3.95–4.16 (6 H, m), 6.30 (1 H, br. s.), 6.76–6.87 (5 H, m), 6.97 (1 H, d, *J =* 8), 7.09–7.21 (5 H, m). ^13^C-NMR: 14.7, 16.1, 18.3, 20.7, 23.7 (2C), 24.9, 29.6, 33.0, 34.5, 36.7, 37.6, 38.1, 38.9, 45.1, 46.9, 65.8, 67.0, 68.5, 80.0, 114.0 (4C), 123.5, 123.7, 126.5, 128.8, 129.7 (2C), 130.0 (2C), 130.5, 134.3, 145.3, 146.6, 156.8, 157.2, 172.2, 178.3. Found: *m*/*z* 655.3873 [M]+. C_41_H_53_NO_6_. Calculated: M 655.3872.

(2S)-3-(4-{2-[4-(2-{[(1R,4aR,10aR)-1,4a-dimethyl-7-(propan-2-yl)-1,2,3,4,4a,4b,5,6,10,10a-decahydrophenanthren-1-yl]formamido}ethoxy)phenyl]ethoxy}phenyl)-2-ethoxypropanoic acid, **9c**.

Yellow oil, 83% yield. ^1^H-NMR: 0.75–0.82 (3 H, m), 0.91–1.02 (5 H, m), 1.08–1.28 (9 H, m), 1.40–1.61 (4 H, m), 1.61–2.09 (9 H, m), 2.90–3.05 (4 H, m), 3.32–3.46 (1 H, m), 3.50–3.71 (3 H, m), 3.94–4.04 (3 H, m), 4.08 (2 H, t, *J =* 7.0), 5.23 (1 H, d, *J =* 3.9), 5.70 (1 H, s), 6.21 (1 H, t, *J =* 5.3), 6.81 (4 H, t, *J =* 9.2), 7.15 (4 H, dd, *J =* 15.7). ^13^C-NMR: 14.5, 15.3, 17.2, 18.5, 21.1, 21.7, 22.7, 24.3, 25.5, 27.6, 30.0, 34.8, 35.2, 37.6, 38.8, 39.5, 45.9, 46.6, 51.2, 61.0, 66.5, 69.0, 80.7, 114.6 (2C), 114.9 (2C), 120.7, 122.6, 129.6, 130.3 (2C), 130.7 (2C), 131.2, 135.7, 145.5, 157.5, 157.8, 172.8, 178.9. Found: *m*/*z* 657.4029 [M]+. C_41_H_55_NO_6_. Calculated: M 657.4028.

(2S)-3-(4-{2-[4-(2-{[(1R,4aR,5S)-5-[2-(furan-3-yl)ethyl]-1,4a-dimethyl-6-methylidene-decahydronaphthalen-1-yl]formamido}ethoxy)phenyl]ethoxy}phenyl)2-ethoxypropanoic acid, **9d**.

Yellow oil, 79% yield. ^1^H-NMR: 0.5 (3 H, s), 0.9–1.3 (11 H, m), 1.4–1.9 (8 H, m), 2.1–2.3 (1 H, m), 2.3–2.6 (2 H, m), 2.8–3.1 (4 H, m), 3.4–3.5 (1 H, m), 3.5–3.7 (3 H, m), 3.9–4.2 (6 H, m), 4.5 (1 H, s), 4.8 (1 H, s), 6.1 (1 H, br. s.), 6.2 (1 H, s), 6.8 (4 H, d, *J =* 7.6), 7.1–7.2 (5 H, m), 7.3 (1 H, s). ^13^C-NMR: 14.9, 15.8, 20.8, 24.2, 24.9, 27.3, 30.8, 35.4, 38.9, 39.1, 39.5, 39.6, 40.0, 41.0, 44.8, 55.8, 57.2, 66.9, 67.1, 69.4, 81.0, 107.3, 111.6, 114.9 (2C), 115.1 (2C), 126.0, 129.9, 130.7 (2C), 131.0 (2C), 131.5, 139.4, 143.3, 148.2, 157.8, 158.2, 173.2, 177.5. Found: *m*/*z* 671.3822 [M]+. C_41_H_53_NO_7_. Calculated: M 671.3822.

### 2.2. Biology

#### 2.2.1. Animals

Male C57BL/6^Ay^ mice weighing 28–32 g were used. Animals were obtained from the Specific Pathogen Free (SPF) vivarium of the ICG SB RAS. The animals were housed in plastic cages with free access to feed and water. In the vivarium, the humidity, temperature, and 12/12 h light-and-dark cycle were controlled. All animal experiments were performed in accordance with the Russian Federation’s laws, the Ministry of Health of the Russian Federation decree no. 199n of 4 January 2016; the European Parliament and European Union Council Directive 2010/63/EU of 22 September 2010 on the protection of animals used for scientific purposes. The experimental protocol was approved by the Ethics Committee of NIOCH SB RAS (protocol no. P-01-04.2022-14).

#### 2.2.2. The OGTT

Mice were fasted for 12 h before the test. Compounds **7a,b, 9a–d** were administered orally at a dose of 30 or 20 mg/kg (according to molar mass) in a Tween-80–water suspension. Glucose was given orally at a dose of 2.5 g/kg. Metformin (MF, CAS 1115-70-4 Acros Organics, Geel, Belgium) was used as a positive control at a dose of 250 mg/kg. OGTT was conducted on the 14th and 28th days of the experiment. During the first OGTT, all compounds were introduced by oral gavage 30 min prior to the glucose load. In the second OGTT, the introduction of the last compounds was a day prior to the test. All mice blood samples were collected by tail incision 0 (before dosing), 30, 60, 90, and 120 min after the glucose load. The ONE TOUCH Select blood glucose meter (LIFESCAN Inc., Milpitas, CA, USA) was used for blood glucose concentration measurements. The area under the glycemic curve (AUC) was calculated using Tai’s model [10].

#### 2.2.3. The ITT

The test was performed after a 4 h fasting on all AY mice in the experiment. Tested compounds were introduced orally 4 h before the insulin injection. Insulin (soluble human insulin, Medsynthesis Plant, Novouralsk, Russia) at a dose of 5 ED/kg was injected i.p. Blood samples were obtained from tail incision at 0 (before dosing), 15, 30, 45, 60, and 90 min after the insulin injection. Blood glucose concentration was evaluated similar to OGTT.

#### 2.2.4. The AY Mice Experiment Design

In order to facilitate body weight gain, mice, in addition to standard chow, were fed with lard and cookies for 30 days. Mice with body weight ≥35 g were chosen for further experiments. Animals were divided into groups of six mice each: (1) Vehicle (water + 2 drops of Tween-80); (2–6) **7a,b**, **9a–d** 30 or 20 mg/kg (according to molar mass); and (7) MF 250 mg/kg. C57BL/6 mice (*n* = 6) + vehicle served as an intact control group (N8). The diet stayed the same throughout the experiment. The tested compounds were administered orally once a day. OGTT was performed as described above. ITT was conducted on the 29th day of the experiment. At the end of the experiment (day 31), the animals were decapitated and blood was collected for the biochemical assay. The following tissues were taken for the histological studies: liver, interscapular white and brown fat, pancreas. Gonadal white fat was taken instead of interscapular fat in the C57Bl/6 mice. Food consumption and body weight were evaluated once a week.

#### 2.2.5. Biochemical Assays

Blood was centrifuged at 1640× *g* for 15 min for serum separation. A photometer Multiscan Ascent (Thermo Labsystems, Helsinki, Finland) and Standard Kits (Vector-Best, Novosibirsk, Russia) were used to analyze the total cholesterol, triglycerides, alanine aminotransferase, and lactate levels in the serum.

#### 2.2.6. Histological Examination

Tissue samples were fixed in 10% neutral buffered formalin for 7 days. After that, they were subjected to the standard dehydration in ascending ethanol concentrations and xylene. The samples were then embedded in paraffin on an AP 280 workstation using Histoplast (Thermo Fisher Scientific, Waltham, MA, USA, melting point of 58 °C). Slices were obtained on a rotational microtome NM 335E with a thickness of 4.5 μm. Hematoxylin and eosin, periodic acid–Schiff, and orange G staining were used. Tissue samples were examined with a light microscope at a magnification of ×100–400.

#### 2.2.7. Body Temperature Measurement

The body temperature was measured using a FLIR C3-X thermal camera (FLIR, Taby, Sweden) at a room temperature of 23 °C. Each animal was placed into the plastic cage and the thermal image was taken. All images were then analyzed using FLIR Thermal Studio Software. The temperature value was taken from a point on the middle of the animal’s back. Results are represented as a group average.

#### 2.2.8. Statistical Analysis

Statistical analysis was performed by the Mann–Whitney U test. Data are shown as the mean ± SEM. Data with *p* < 0.05 were considered statistically significant.

## 3. Results

### 3.1. Chemistry

**Scheme 1:**
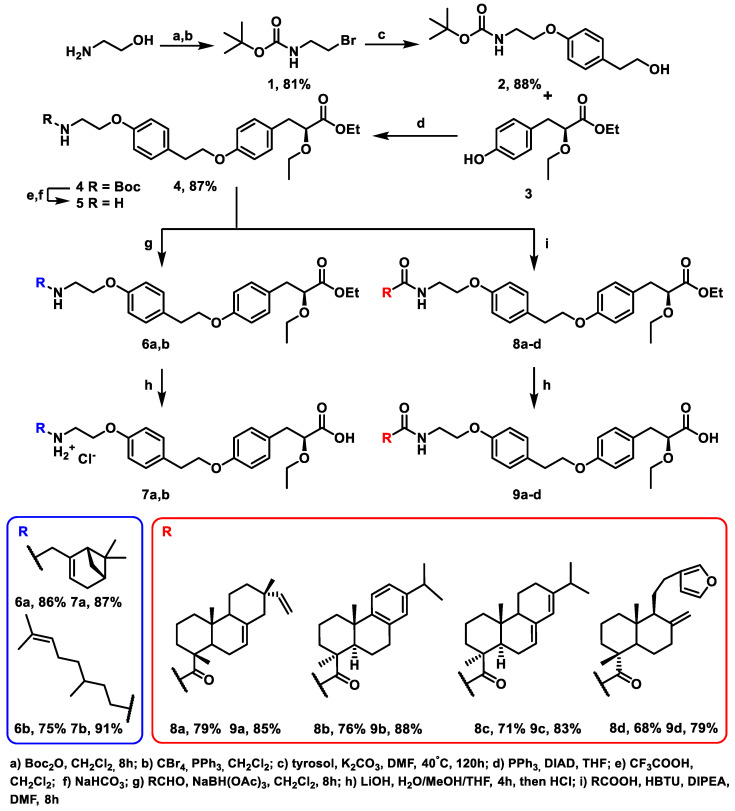
Synthesis of the target compounds.

For the synthesis of the scaffold, which was common for all the target compounds, we used the synthesis technique described earlier [8], where aminoethanol was chosen as the starting material (Scheme 1). Bromide **1** was prepared in two steps: protection of the amino group by di-tert-butyl bicarbonate followed by the Appel bromination reaction in the presence of CBr_4_ and PPh_3_ in methylene chloride. Further interaction of bromide **1** with tyrosol by the nucleophilic substitution reaction (S_N_2) in the presence of K_2_CO_3_ in DMF resulted in the formation of alcohol **2**. Ether **4** was obtained by the interaction of alcohol **2** with phenol **3** by the Mitsunobu reaction in the presence of diisopropyl azodicarboxylate (DIAD) and PPh_3_ in THF. Free amine **5** was obtained in a 92% yield after sequential treatment of trifluoroacetic acid on amine **4** within the methylene chloride and aqueous NaHCO_3_ solution.

It was shown that amine **5** reacts with terpenoids citronellal and (+)-myrtenal by the reductive amination reaction in the presence of triacetoxyborohydride in methylene chloride to form a secondary amine with excellent yields of 75% and 86% after column chromatography. In order to inhibit the possibility of the formation of a tertiary amine in the subsequent addition of aldehyde to the secondary amine formed, we used a 5% amine excess.

Condensation of the obtained amine **5** with diterpene acids was performed under solid phase peptide synthesis conditions in the presence of HBTU and DIPEA in N,N-dimethylformamide (DMF). Using this approach, the target amides **8a–d** were isolated in high yields of 68–79% after column chromatography.

Hydrolysis of the ester group of compounds **6a,b** and **8a–d** was carried out under previously selected conditions described earlier [8]. The use of lithium hydroxide in the methanol–tetrahydrofuran–water mixture led to the hydrolysis of the ester group in compounds **8a–d** under mild conditions in a short period of time. As a result, after acidic treatment of the reaction mixture, the desired acids were obtained in good yields of 79–88% with no further purification. The ester group of monoterpene derivatives **6a,b** were hydrolyzed under similar conditions; after acidic treatment of the reaction mixture, the target compounds were isolated as acid hydrochlorides **7a,b** in yields of 87–91%.

### 3.2. Biology

All the synthesized substances were studied in obese mice with impaired glucose tolerance (C57Bl/6^Ay^ line, AY mice). Among them, only compound **9a** showed a significant pharmacological effect, and therefore, all the results below will be presented only for this substance.

#### 3.2.1. Body Weight and Feed Intake

**Figure 3:**
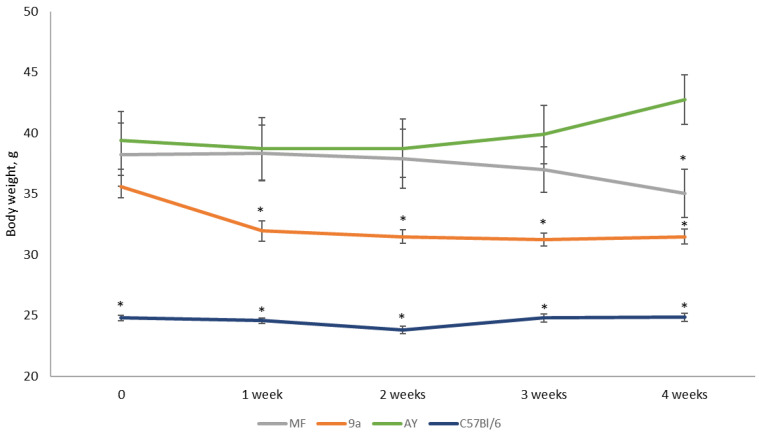
Body weight change during the experiment. * *p* < 0.05 compared to the AY mice.

During the first 2 weeks of the experiment, all animals showed a decrease in body weight, which can be attributed to the stress of the daily administration of substances, then in AY mice (negative control), the body weight began to increase and by the end of the experiment, was higher than at the beginning. In the C57Bl/6 mice (intact control), it returned to the baseline by the fourth week of the experiment. Mice receiving metformin (positive control) and **9a** showed a steady decrease in body weight by the end of the experiment (Figure 3). It is worth noting that the feed intake of mice in group **9a** was at the same level as that of mice in group AY, whereas mice receiving metformin consumed more feed in the first two weeks (Figure 3 and Table 1).

#### 3.2.2. OGTT at Weeks 2 and 4 of the Experiment

**Figure 4:**
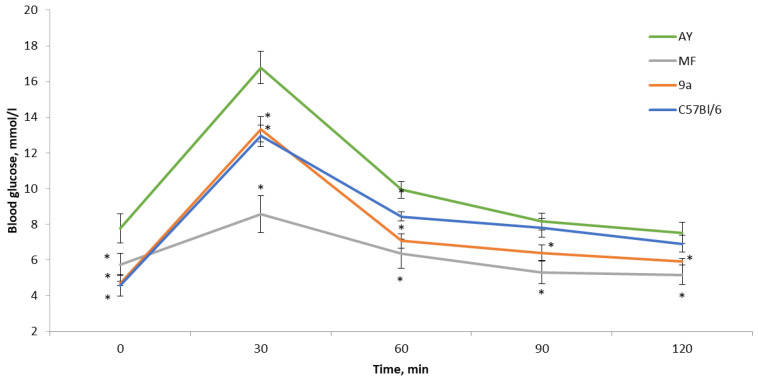
OGTT results. The test conducted after 14 days of AY mice treatment by compound **9a**. Doses: **9a**–30 mg/kg, MF–250 mg/kg. * *p* < 0.05 compared to the AY mice.

The first study of glucose tolerance in mice was performed after 14 days of **9a** administration. A significant hypoglycemic effect was observed, which, however, was inferior to that of metformin (Figure 4).

**Figure 5:**
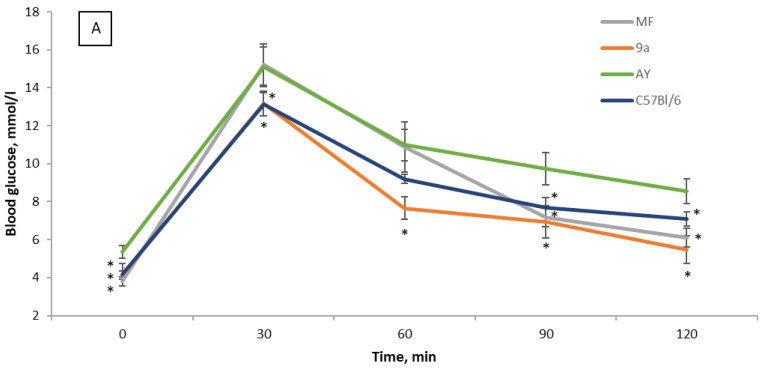

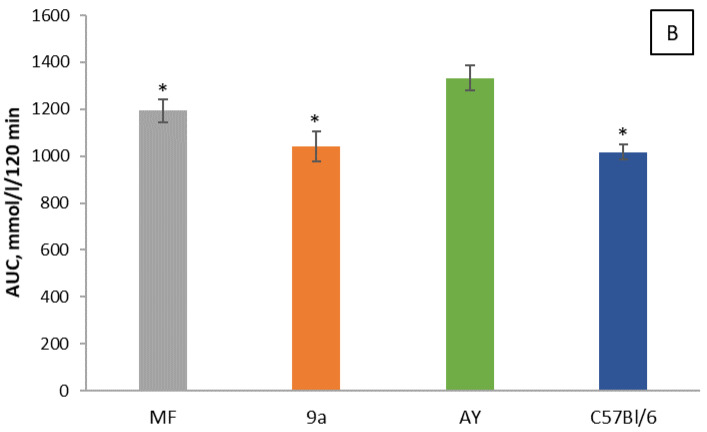
(**A**) OGTT results. The test conducted after 28 days of AY mice treatment using compound **9a**. (**B**) AUC calculated according to the OGTT data after 28 days of AY mice treatment using compound **9a**. Doses: **9a**–30 mg/kg, MF–250 mg/kg. * *p* < 0.05 compared to the AY mice.

The second OGTT was performed after 28 days of **9a** administration, and unlike the previous test, the studied substances were not administered 30 min before glucose load, but the cumulative effect was evaluated for the entire duration of the experiment. As can be seen from Figure 5A, the greatest hypoglycemic effect was detected in mice treated with **9a**, while the effect of metformin was not evident until 90 min after glucose administration.

#### 3.2.3. ITT at the End of the Experiment

**Figure 6:**
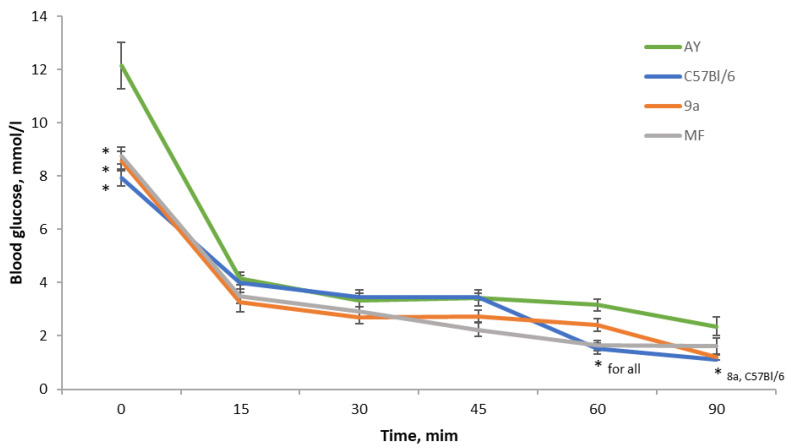
Blood glucose levels in ITT performed 29 days after the beginning of the experiment. * *p* < 0.05 compared to the AY mice.

At the end of the experiment, an ITT was performed to determine the sensitivity of the mice to insulin. As can be seen from Figure 6, in all animals, insulin administration led to a marked decrease in blood glucose levels, but only in the intact control and in the **9a** mice, it fell to 1.1 mmol/L, which was the detection limit of the glucometer.

#### 3.2.4. Body Temperature

**Figure 7:**
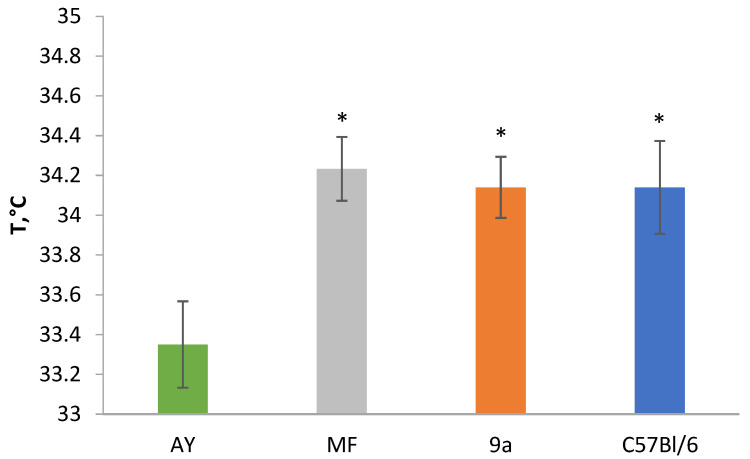
Body temperature of the mice at the end of the experiment. * *p* < 0.05 compared to the AY mice.

At the end of the experiment, we measured the body temperature of the animals in a non-invasive way. It was found that the administration of **9a** and metformin significantly increased the body temperature of the mice, aligning it with the value for the C57Bl/6 mice (Figure 7).

#### 3.2.5. Evaluation of Biochemical Parameters of Blood and Tissue Mass

At the end of the experiment, the weight of the liver and adipose tissue was assessed in the animals. It was found that the mass of the liver in the **9a** group did not differ from that of the AY mice of the other groups. In contrast, the mass of white and brown adipose tissue was significantly lower (Table 2).

The biochemical blood analysis demonstrated a decrease in the lactate and ALT levels in mice treated with **9a** (Table 3).

#### 3.2.6. Histological Analysis

**Figure 8:**
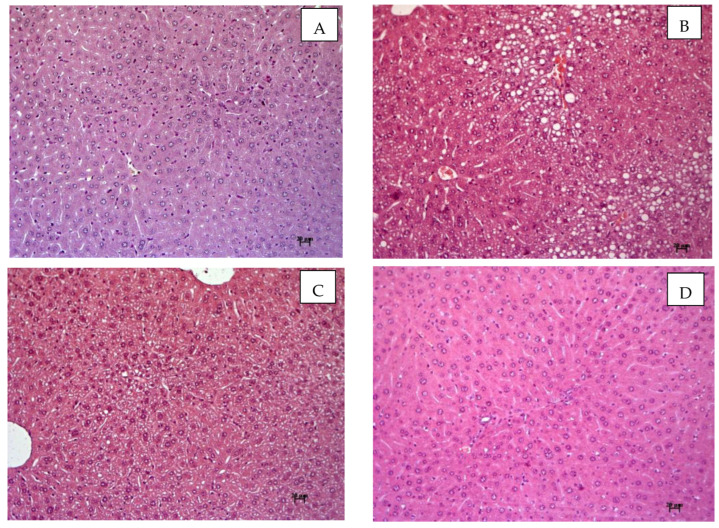
Histological evaluation of the liver in mice after 4 weeks of experiment. (**A**) AY mice treated by compound **9a** at a dose of 30 mg/kg, (**B**) AY mice (untreated), (**C**) AY mice treated by metformin at a dose of 250 mg/kg, (**D**) C57Bl/6 (healthy control). Hematoxylin and eosin staining, magnification ×200.

**Figure 9:**
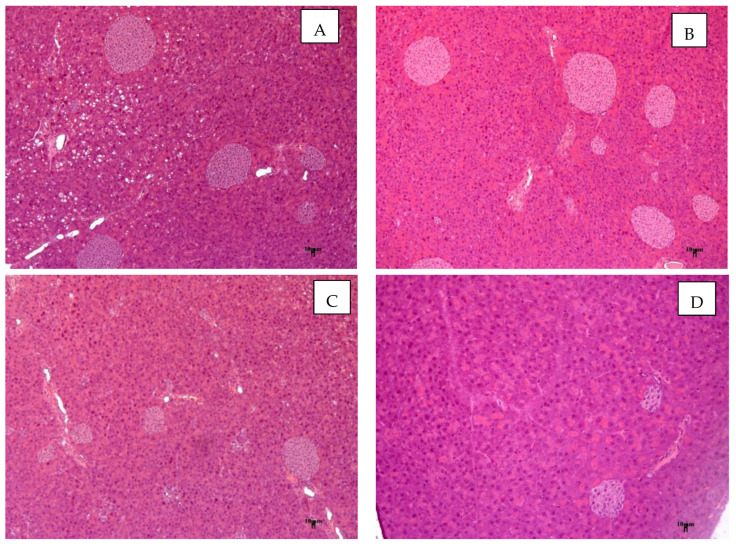
Histological evaluation of pancreas in mice after 4 weeks of experiment. (**A**) AY mice treated by compound **9a** at a dose of 30 mg/kg, (**B**) AY mice (untreated), (**C**) AY mice treated by metformin at a dose of 250 mg/kg, (**D**) C57Bl/6 (healthy control). Hematoxylin and eosin staining, magnification ×100.

**Figure 10:**
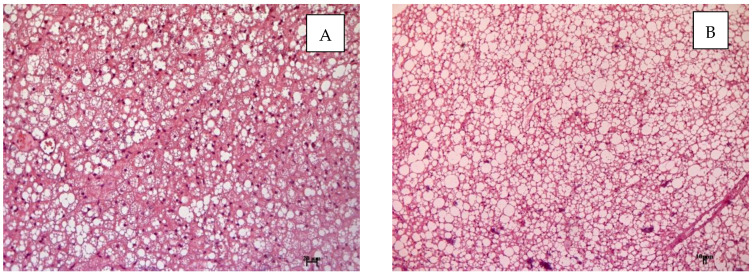

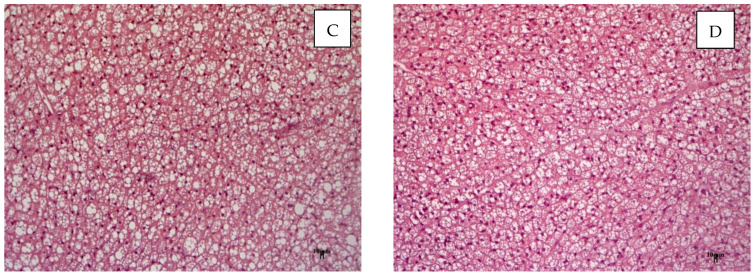
Histological evaluation of brown fat in mice after 4 weeks of experiment. (**A**) AY mice treated by compound **9a** at a dose of 30 mg/kg, (**B**) AY mice (untreated), (**C**) AY mice treated by metformin at a dose of 250 mg/kg, (**D**) C57Bl/6 (healthy control). Hematoxylin and eosin staining, magnification ×100.

**Figure 11:**
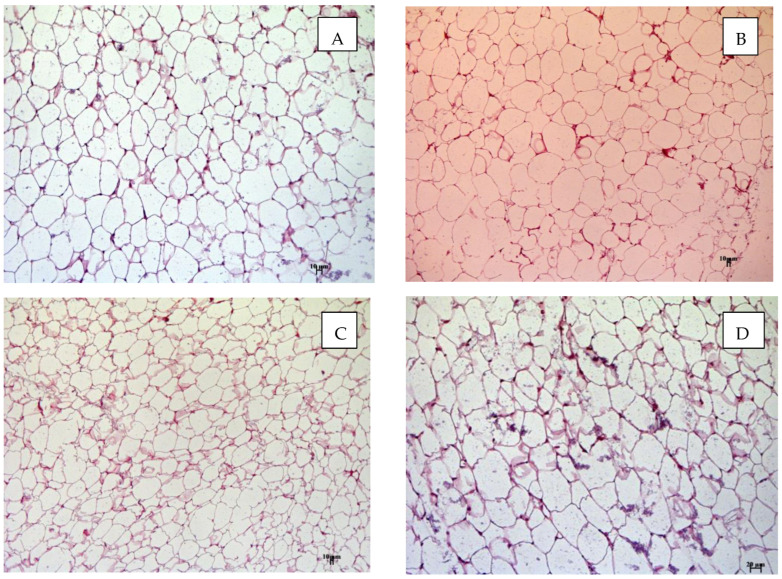
Histological evaluation of brown fat in mice after 4 weeks of experiment. (**A**) AY mice treated by compound **9a** at a dose of 30 mg/kg, (**B**) AY mice (untreated), (**C**) AY mice treated by metformin at a dose of 250 mg/kg, (**D**) C57Bl/6 (healthy control). Hematoxylin and eosin staining, magnification ×100.

Histological examination revealed fatty hepatosis (Figure 8B) and pronounced hyperplasia of islet apparatus in the endocrine part of the pancreas (Figure 9B) in AY mice. Brown adipose tissue examination revealed a marked increase in the adipocyte fat content and fat cysts formed from large fat droplets could be found (Figure 10B). The adipocyte size was also dramatically increased in the white adipose tissue (Figure 11B). These metabolic abnormalities were improved in mice treated with metformin (Figure 8C, Figure 9C, Figure 10C and Figure 11C).

In animals treated with **9a**, the morphological picture in the studied organs practically corresponds to that in the group of intact animals. Architectonics of the liver and pancreas had typical structure, and no pronounced infiltrative-necrotic, hemodynamic changes were found (Figure 8A and Figure 9A). In brown and white adipose tissue, the fat content in adipocytes was comparable with the intact group (Figure 10A and Figure 11A).

## 4. Discussion

In this work, we synthesized several new mono- and diterpenic derivatives with a pharmacophore fragment of *(S)*-2-ethoxy-3-phenylpropanoic acid, which is common to glitazars.

**Figure 12:**
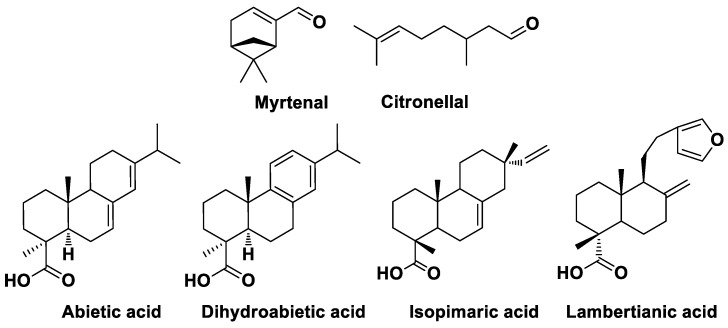
Monoterpenoids and diterpenic acids.

The choice of monoterpenoids for derivatization was also made based on the literature data on the hypoglycemic and hypolipidemic activity of the terpene derivatives. Derivatives of both bicyclic and acyclic monoterpenoids (Figure 12) are known to be partial PPAR agonists and also have hypoglycemic and hepatoprotective activity [11,12,13,14].

Abietic, dehydroabietic, isopimaric, and lambertianic acids (Figure 12) were chosen for the synthesis due to their availability and their own biological properties. Among the diterpene acids, abietane type acids are known to influence glucose levels as well as the lipid profile [15,16]. Lambertianic acid has anti-inflammatory as well as potential antidiabetic activity [17].

Isopimaric acid (IPA) has a wide range of biological activity. It has been found to exhibit marked antitumor [18], antibacterial [19,20], and anti-inflammatory [21,22] activities. It is worth noting that IPA amides were shown to have a weak cytotoxicity against the GepG2 cell lines [23]. IPA amides also exhibit an analgesic effect on male inbred mice [24]. Moreover, IPA was shown to inhibit protein-tyrosine phosphatase 1B (PTP1B) better than abietic and dehydroabietic acid [25]. PTP1B is an enzyme in the protein tyrosine phosphatase (PTP) family that is responsible for the regulation of many processes, particularly metabolism, and often contributes to diseases that occur when these processes are disrupted (diabetes, cancer, autoimmune, and Alzheimer’s diseases) [25].

All synthesized compounds were studied in mice with obesity and impaired glucose tolerance (C57Bl/6^Ay^). In these mice, agouti gene (Ay/a) mutation resulted in antagonism of melanocortin receptors by the agouti protein and in turn led to the emergence of yellow pigmentation, late-onset obesity, and hyperinsulinemia [26]. Such metabolic abnormalities make AY mice a convenient animal model for examining the effects on glucose and lipid metabolism. Among the studied substances, we found one pharmacologically active compound **9a**, which is a derivative of isopimaric acid. Its pharmacological effects in our animal experiments are characteristic of the action of PPAR-alpha and gamma agonists. First, a significant reduction in fatty hepatosis and a decrease in the weight of white and brown adipose tissue were demonstrated. All of this indicates an acceleration of triglyceride catabolism occurring upon the activation of PPAR-alpha [27]. In brown adipose tissue, increased catabolism of fatty acids usually results in increased heat production due to the activation of uncoupling protein 1 (UCP1) [28], which was demonstrated by the increase in the body temperature of the mice at the end of the experiment (Figure 7). However, despite all of these changes, we did not find a decrease in the level of TG in the animals’ blood, perhaps a longer administration of the studied substance could have led to a decrease in this parameter. Second, PPAR-gamma activation is usually associated with increased tissue sensitivity to the action of insulin, and as a consequence, a decrease in the level of glycemia [29]. The administration of compound **9a** resulted in a marked decrease in both the fasting glucose levels and significantly improved the glucose tolerance of the mice (Figure 4 and Figure 5). The most pronounced effect was achieved after four weeks of administration. This was due to the increased sensitivity of animal tissues to insulin, which is further confirmed by the results of ITT (Figure 6) and a decrease in the blood lactate levels (Table 3). Lactate in obesity is in large quantities synthesized and excreted into the blood by fat cells and its elevated levels are associated with insulin resistance. The decrease in its blood level can be considered as further evidence of improved tissue sensitivity to insulin [30]. Histological examination data, liver weight, and biochemical blood tests (decreased ALT level, Table 3) demonstrated the absence of toxic liver damage caused by compound **9a**, which was the reason for at least the discontinuation of Imiglitazar’s clinical studies [31]. Of course, it is too early to say whether compound **9a** is safe because additional experiments are needed, since the most typical side effects of dual PPAR agonists are cardiac and renal damage [31].

It is also interesting to note that the derivatives of these acids, which themselves have hypoglycemic and/or hypolipidemic effects, did not show such an effect in our experiments.

## 5. Conclusions

Compounds **7a,b** and **9a–d**, incorporating various diterpenic acids as well as monoterpenoid fragments in their structure as “tails”, were synthesized. The study of their pharmacological activity in obese and DM2 (C57Bl/6^Ay^) mice revealed one compound, **9a** (isopimaric acid derivative), having effects characteristic for PPAR-alpha and gamma dual agonists. Its administration at a dose of 30 mg/kg for one month led to a decrease in triglyceride levels in the liver and adipose tissue of mice by increasing their catabolism and to a hypoglycemic effect associated with an improvement in the insulin sensitivity of the mouse tissue. In addition, the substance was shown to have no toxic effects on the liver. To the best of our knowledge, compound **9a** is the first example among isopimaric acid derivatives with the hypoglycemic and hypolipidemic activities demonstrated in vivo. The synthesis of other isopimaric acid derivatives seems promising to reveal the structure–hypoglycemic/hypolipidemic activity relationship.

## Figures and Tables

**Table 1:** Food consumption by mice during the experiment. Data represented as food intake (total amount of standard chow, cookies, and lard) in g per a mouse in the group.

	MF	9a	AY	C57Bl/6
1 week	29.9	23.5	23.7	23.2
2 weeks	29.8	23.9	26.2	23.6
3 weeks	25.9	24.5	25.3	23.4
4 weeks	26.2	23.4	23.8	23.8

**Table 2:** Mass of livers, gonadal fat pads, interscapular fat pads, and brown fat in the AY mice treated for four weeks by compound **9a** at a dose of 30 mg/kg and MF at a dose of 250 mg/kg.

	Body Mass, g	Liver Mass, g	Gonadal Fat Pad, g	Interscapular Fat Pad, g	Interscapular Brown Fat, g
C57Bl/6	21.90 ± 0.30 *	0.85 ± 0.013 *	0.27 ± 0.02 *	-	0.06 ± 0.003 *
AY	36.97 ± 2.41	1.09 ± 0.05	1.99 ± 0.26	1.10 ± 0.16	0.20 ± 0.009
**9a**	27.86 ± 0.49	1.00 ± 0.05	1.14 ± 0.14 *	0.38 ± 0.03 *	0.10 ± 0.006 *
MF	32.45 ± 2.01	1.07 ± 0.06	1.93 ± 0.26	0.73 ± 0.12	0.13 ± 0.02 *

* *p* < 0.05 compared to the AY mice.

**Table 3:** Biochemical parameters of mouse blood.

Group	TC, mmol/L	TG, mmol/L	Lactate, mmol/L	ALT, U/L
C57Bl/6	4.11 ± 0.03	1.37 ± 0.02 *	8.94 ± 0.29	13.39 ± 3.36 *
AY	4.03 ± 0.05	1.54 ± 0.03	8.63 ± 0.11	20.25 ± 3.10
**9a**	3.99 ± 0.05	1.54 ± 0.04	7.33 ± 0.48 *	9.89 ± 1.16 *
MF	4.48 ± 0.48	1.53 ± 0.09	7.93 ± 0.24 *	15.71 ± 1.50

TC—total cholesterol, TG—triglycerides, TP—total protein, ALT—alanine aminotransferase, AST—aspartate transaminase. * *p* < 0.05 compared to the AY mice.

## Data Availability

The data presented in this study are available on request from the corresponding authors.

## Supplementary Materials

The following supporting information can be downloaded at: https://www.mdpi.com/article/10.3390/cimb45030144/s1. Figures S1–S24—1H and 13C NMR spectra of compound **6a-9d**.

## References

[1] Rochlani Y., Pothineni N.V., Kovelamudi S., Mehta J.L. (2017). Metabolic syndrome: Pathophysiology, management, and modulation by natural compounds. Ther. Adv. Cardiovasc. Dis..

[2] Oladejo A.O. (2011). Overview of the metabolic syndrome; an emerging pandemic of public health significance. Ann. Ib. Postgrad. Med..

[3] Hong F., Xu P., Zhai Y. (2018). The Opportunities and Challenges of Peroxisome Proliferator-Activated Receptors Ligands in Clinical Drug Discovery and Development. Int. J. Mol. Sci..

[4] Varga T., Czimmerer Z., Nagy L. (2011). PPARs are a unique set of fatty acid regulated transcription factors controlling both lipid metabolism and inflammation. Biochim. Biophys. Acta (BBA)—Mol. Basis Dis..

[5] Agrawal R. (2014). The First Approved Agent in the Glitazar’s Class: Saroglitazar. Curr. Drug Targets.

[6] Balakumar P., Mahadevan N., Sambathkumar R. (2019). A Contemporary Overview of PPARα/γ Dual Agonists for the Management of Diabetic Dyslipidemia. Curr. Mol. Pharmacol..

[7] Xi Y., Zhang Y., Zhu S., Luo Y., Xu P., Huang Z. (2020). PPAR-Mediated Toxicology and Applied Pharmacology. Cells.

[8] Fomenko V., Blokhin M., Kuranov S., Khvostov M., Baev D., Borisova M.S., Luzina O., Tolstikova T.G., Salakhutdinov N.F. (2021). Triterpenic Acid Amides as a Promising Agent for Treatment of Metabolic Syndrome. Sci. Pharm..

[9] Larsson M., AstraZeneca (2003). Process for The Preparation of 2-Ethoxy-3-[4-(2-(Methanesulphonyloxyphenyl)-Ethoxy) Phenyl] Propanoic Acid. World Patent.

[10] Tai M.M. (1994). A Mathematical Model for the Determination of Total Area Under Glucose Tolerance and Other Metabolic Curves. Diabetes Care.

[11] Kuranov S., Marenina M., Ivankin D., Blokhin M., Borisov S., Khomenko T., Luzina O., Khvostov M., Volcho K., Tolstikova T. (2022). The Study of Hypoglycemic Activity of 7-Terpenylcoumarins. Molecules.

[12] Srinivasan S., Muruganathan U. (2016). Antidiabetic efficacy of citronellol, a citrus monoterpene by ameliorating the hepatic key enzymes of carbohydrate metabolism in streptozotocin-induced diabetic rats. Chem.-Biol. Interact..

[13] Lu J.-X., Qiu Y., Guo L.-J., Song P., Xu J., Wan G.-R., Wang S.-X., Yin Y.-L., Li P. (2021). Potential therapeutic effect of Citronellal on diabetic cardiomyopathy in experimental rats. Evid.-Based Complement. Altern. Med..

[14] Zhang Y., Wang Y., Li X., Gu K., Li M., Zhang Y., Zhang Z., Wang S., Li Z. (2020). WSF-7 Inhibits Obesity-Mediated PPARγ Phosphorylation and Improves Insulin Sensitivity in 3T3-L1 Adipocytes. Biol. Pharm. Bull..

[15] Xie Z., Gao G., Wang H., Li E., Yuan Y., Xu J., Zhanga Z., Wanga P., Fua Y., Zenga H. (2020). Dehydroabietic acid alleviates high fat diet-induced insulin resistance and hepatic steatosis through dual activation of PPAR-γ and PPAR-α. Biomed. Pharmacother..

[16] Takahashi N., Kawada T., Goto T., Kim C.-S., Taimatsu A., Egawa K., Yamamoto T., Jisaka M., Nishimura K., Yokota K. (2003). Abietic acid activates peroxisome proliferator-activated receptor-γ (PPARγ) in RAW264.7 macrophages and 3T3-L1 adipocytes to regulate gene expression involved in inflammation and lipid metabolism. FEBS Lett..

[17] Lee M.-S., Cho S.-M., Lee M., Lee E.-O., Kim S.-H., Lee H.-J. (2016). Ethanol extract of *Pinus koraiensis* leaves containing lambertianic acid exerts anti-obesity and hypolipidemic effects by activating adenosine monophosphate-activated protein kinase (AMPK). BMC Complement. Altern. Med..

[18] Gromova M.A., Kharitonov Y.V., Pokrovskii M.A., Bagryanskaya I.Y., Pokrovskii A.G., Shul’ts E.E. (2019). Synthetic Transformations of Higher Terpenoids. 37. Synthesis and Cytotoxicity of 4-(Oxazol-2-Yl)-18-Norisopimaranes. Chem. Nat. Compd..

[19] Smith E., Williamson E., Zloh M., Gibbons S. (2005). Isopimaric acid from Pinus nigra shows activity against multidrug-resistant and EMRSA strains of Staphylococcus aureus. Phytother. Res..

[20] Ulubelen A., Öksüz S., Topcu G., Gören A.C., Bozok-johansson C., Çelik C., Kökdi G., Voelter W. (2001). A New Antibacterial Diterpene from the Roots of Salvia caespitosa. Nat. Prod. Lett..

[21] Massaro F.C., Brooks P.R., Wallace H.M., Russell F.D. (2011). Cerumen of Australian stingless bees (*Tetragonula carbonaria*): Gas chromatography-mass spectrometry fingerprints and potential anti-inflammatory properties. Naturwissenschaften.

[22] Gelmini F., Beretta G., Anselmi C., Centini M., Magni P., Ruscica M., Cavalchini A., Maffei Facino R. (2013). GC–MS profiling of the phytochemical constituents of the oleoresin from *Copaifera langsdorffii* Desf. and a preliminary in vivo evaluation of its antipsoriatic effect. Int. J. Pharm..

[23] Liu J.-J., Lu Y.-J., Zhao Z.-D., Xu S.-C., Bi L.-W. (2018). Synthesis and Cytotoxic Activity of Amides from Isopimaric Acid. Chem. Nat. Compd..

[24] Gromova M.A., Kharitonov Y.V., Borisov S.A., Baev D.S., Tolstikova T.G., Shul’ts E.E. (2021). Synthetic Transformations of Higher Terpenoids. 39.∗ Synthesis and Analgesic Activity of Isopimaric Acid Derivatives. Chem. Nat. Compd..

[25] Hjortness M.K., Riccardi L., Hongdusit A., Ruppe A., Zhao M., Kim E.Y., Zwart P.H., Sankaran B., Arthanarie H., Sousaf M.C. (2018). Abietane-Type Diterpenoids Inhibit Protein Tyrosine Phosphatases by Stabilizing an Inactive Enzyme Conformation. Biochemistry.

[26] Qi Y., Takahashi N., Hileman S.M., Patel H.R., Berg A.H., Pajvani U.B., Scherer P.E., Ahima R.S. (2004). Adiponectin acts in the brain to decrease body weight. Nat. Med..

[27] Eldor R., De Fronzo R.A., Abdul-Ghani M. (2013). In Vivo Actions of Peroxisome Proliferator–Activated Receptors: Glycemic control, insulin sensitivity, and insulin secretion. Diabetes Care.

[28] Saito M., Matsushita M., Yoneshiro T., Okamatsu-Ogura Y. (2020). Brown Adipose Tissue, Diet-Induced Thermogenesis, and Thermogenic Food Ingredients: From Mice to Men. Front. Endocrinol..

[29] Janani C., Ranjitha Kumari B.D. (2015). PPAR Gamma Gene—A Review. Diabetes Metab. Syndr. Clin. Res. Rev..

[30] Lovejoy J., Newby F., Gebhart S., DiGirolamo M. (1992). Insulin resistance in obesity is associated with elevated basal lactate levels and diminished lactate appearance following intravenous glucose and insulin. Metabolism.

[31] Kalliora C., Drosatos K. (2020). The Glitazars Paradox: Cardiotoxicity of the Metabolically Beneficial Dual PPARα and PPARγ Activation. J. Cardiovasc. Pharmacol..

