# Supplementary material for: Terpene-Containing Analogues of Glitazars as Potential Therapeutic Agents for Metabolic Syndrome
Source: Curr Issues Mol Biol. 2023 Mar 8;45(3):2230–47. doi: 10.3390/cimb45030144 (PMC10047834; doi:10.3390/cimb45030144)
Supplement: Supplementary file 1 [file cimb-45-00144-s001.zip › cimb-2224935-supplementary.pdf]

Supporting Information for:

Terpene-Containing Analogues of Glitazars as Potential Therapeutic Agents for Metabolic Syndrome

Mikhail E. Blokhin, Sergey O. Kuranov, Mikhail V. Khvostov, Vladislav V. Fomenko, Olga A. Luzina *, Natalia A. Zhukova, Cham Elhajjar, Tatiana G. Tolstikova and Nariman F. Salakhutdinov

N.N. Vorozhtsov Novosibirsk Institute of Organic Chemistry, 630090 Novosibirsk, Russia

* Correspondence: luzina@nioch.nsc.ru

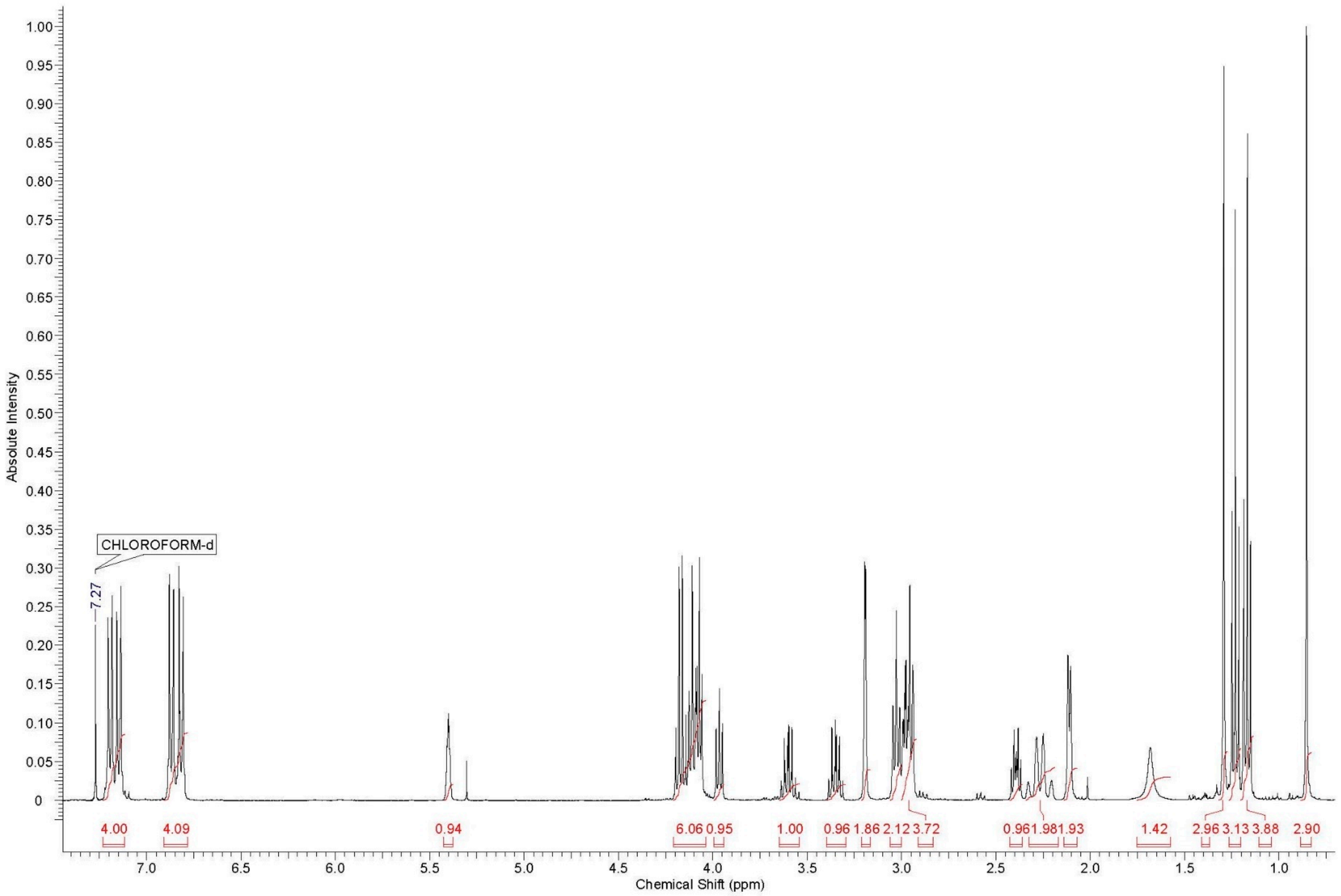

Figure S1. Ethyl (2S)-3-[4-(2-{4-[2-({[(1R,5S)-6,6-dimethylbicyclo[3.1.1]hept-2-en-2-yl]methyl} amino)ethoxy]phenyl}ethoxy)phenyl]-2-ethoxypropanoate (**6a**) ^{1}H NMR spectrum

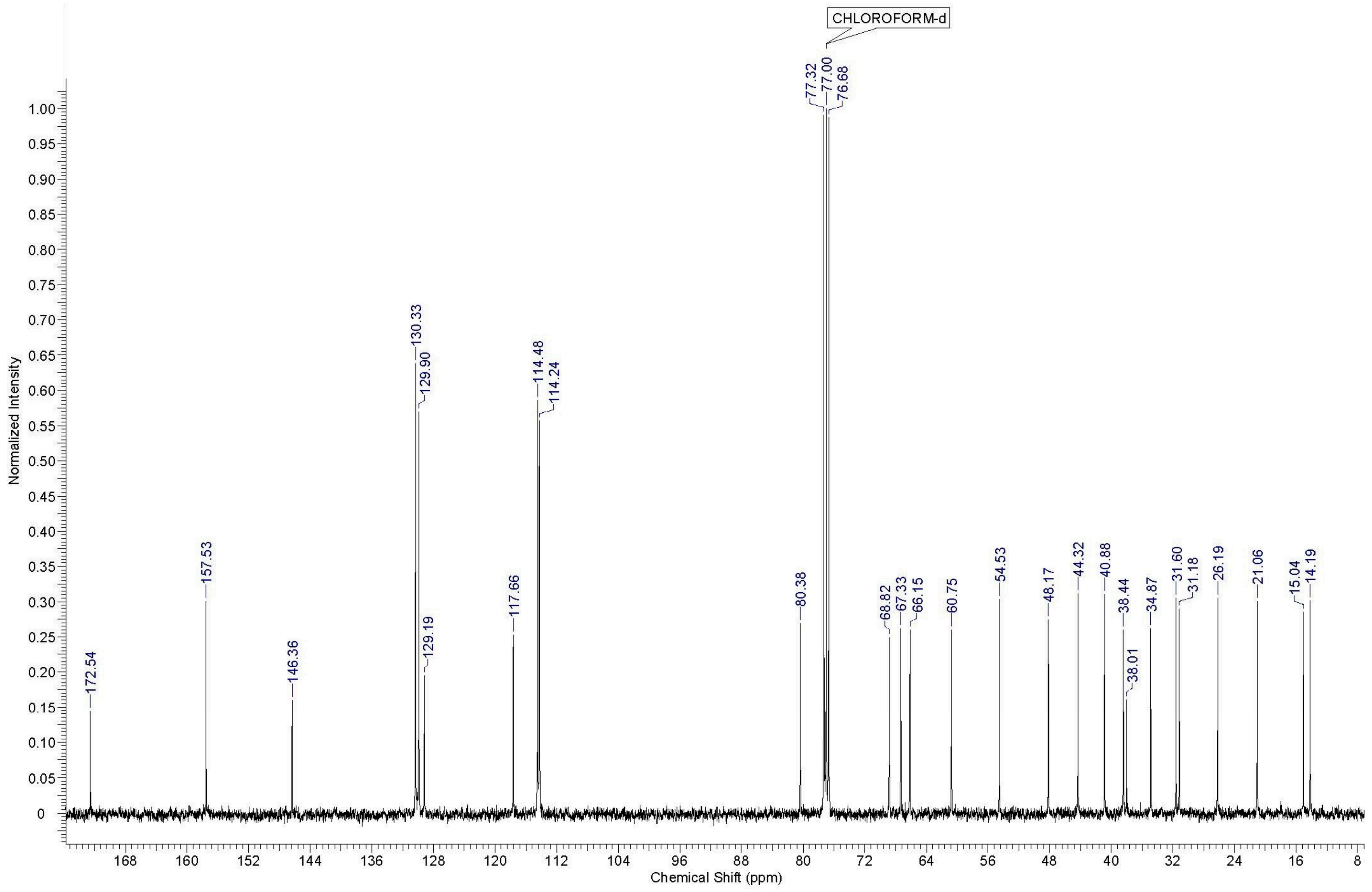

Figure S2. Ethyl (2S)-3-[4-(2-{4-[2-({[(1R,5S)-6,6-dimethylbicyclo[3.1.1]hept-2-en-2-yl]methyl} amino)ethoxy]phenyl}ethoxy)phenyl]-2-ethoxypropanoate (**6a**) ^{13}C NMR spectrum

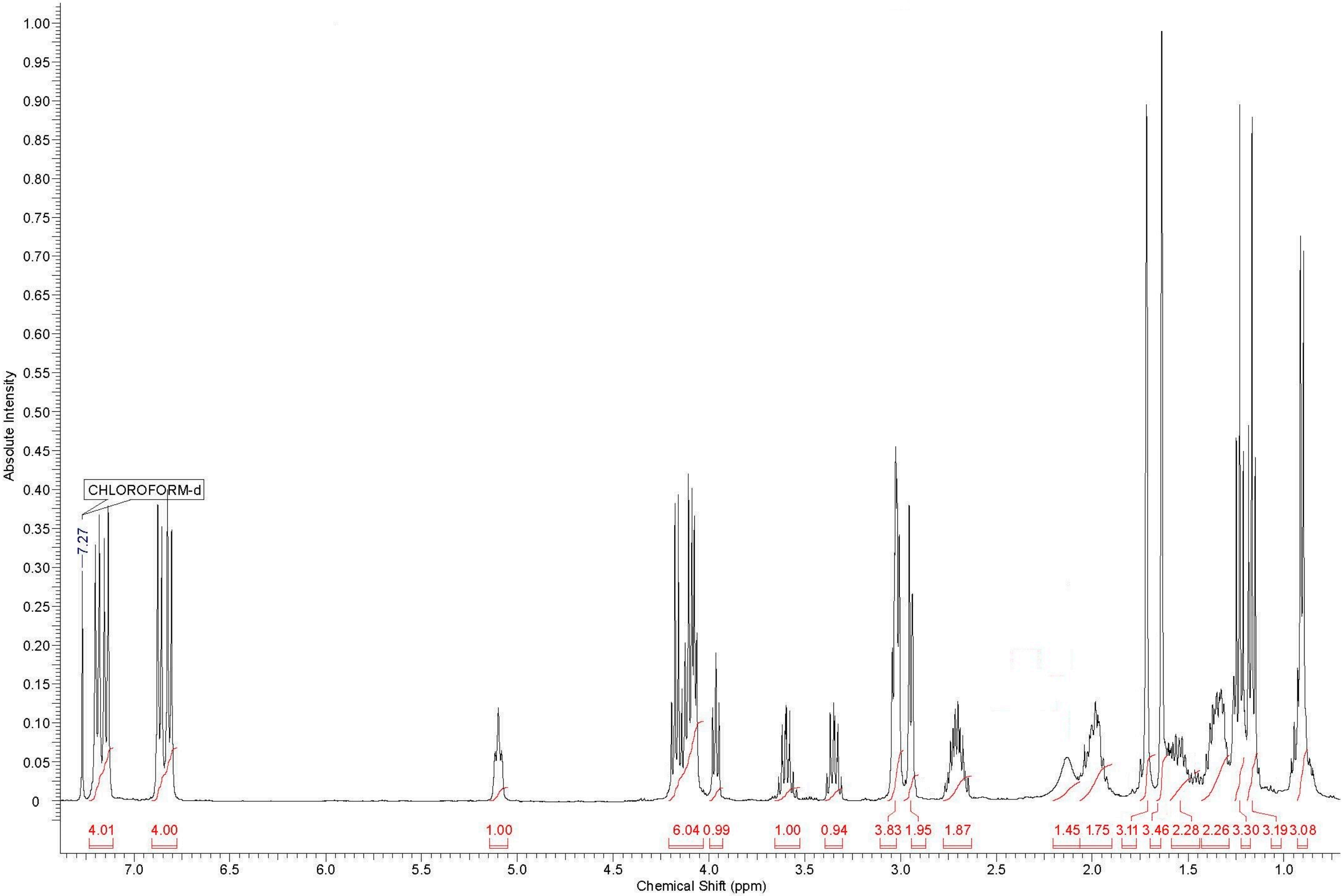

Figure S3. Ethyl (2S)-3-{4-[2-(4-{2-[(3,7-dimethyloct-6-en-1-yl)-amino]-ethoxy}-phenyl)-ethoxy] phenyl}-2-ethoxypropanoate (**6b**) ^{1}H NMR spectrum

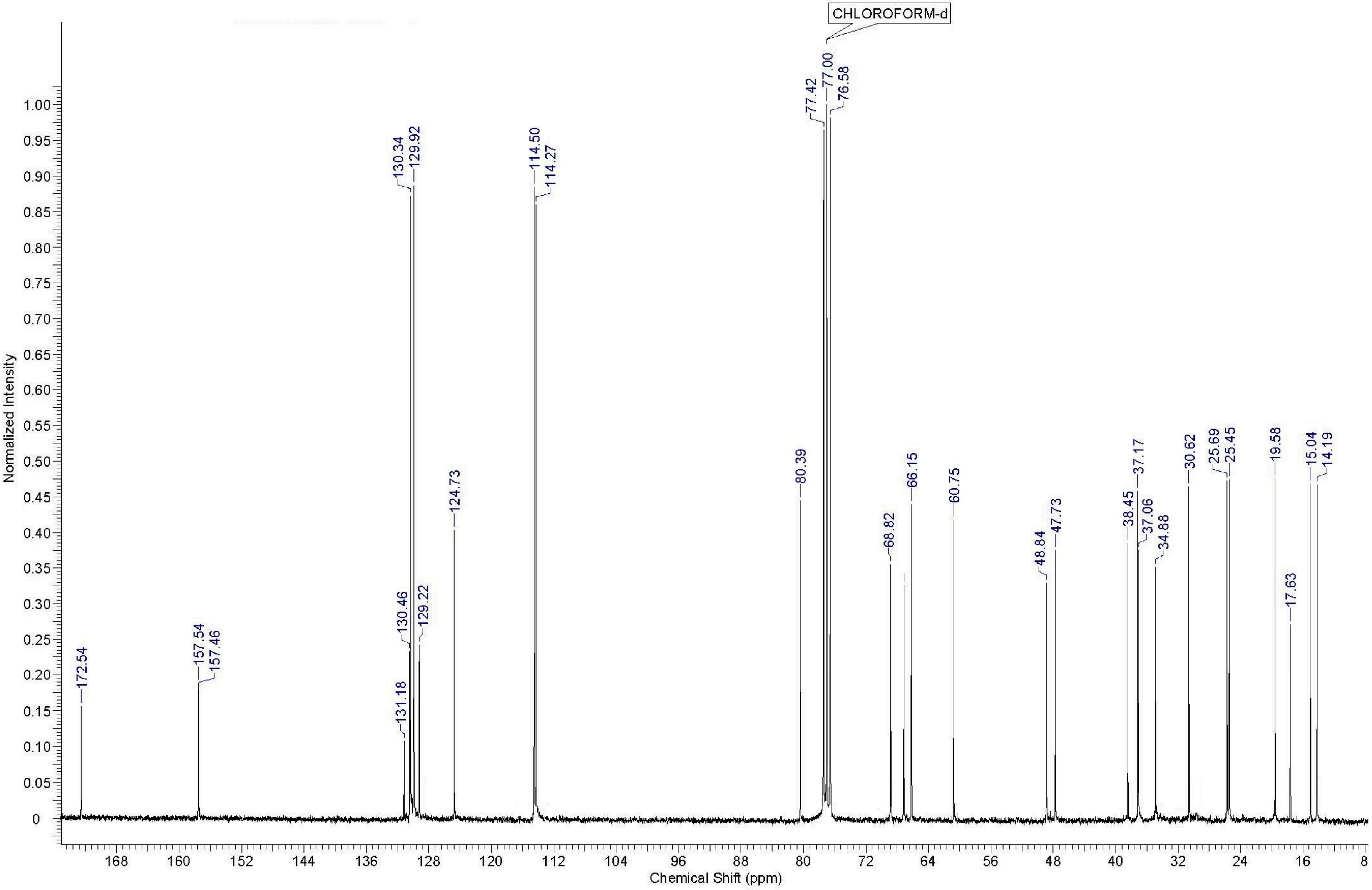

Figure S4. Ethyl (2S)-3-{4-[2-(4-{2-[(3,7-dimethyloct-6-en-1-yl)-amino]-ethoxy}-phenyl)-ethoxy] phenyl}-2-ethoxypropanoate (**6b**) ^{13}C NMR spectrum

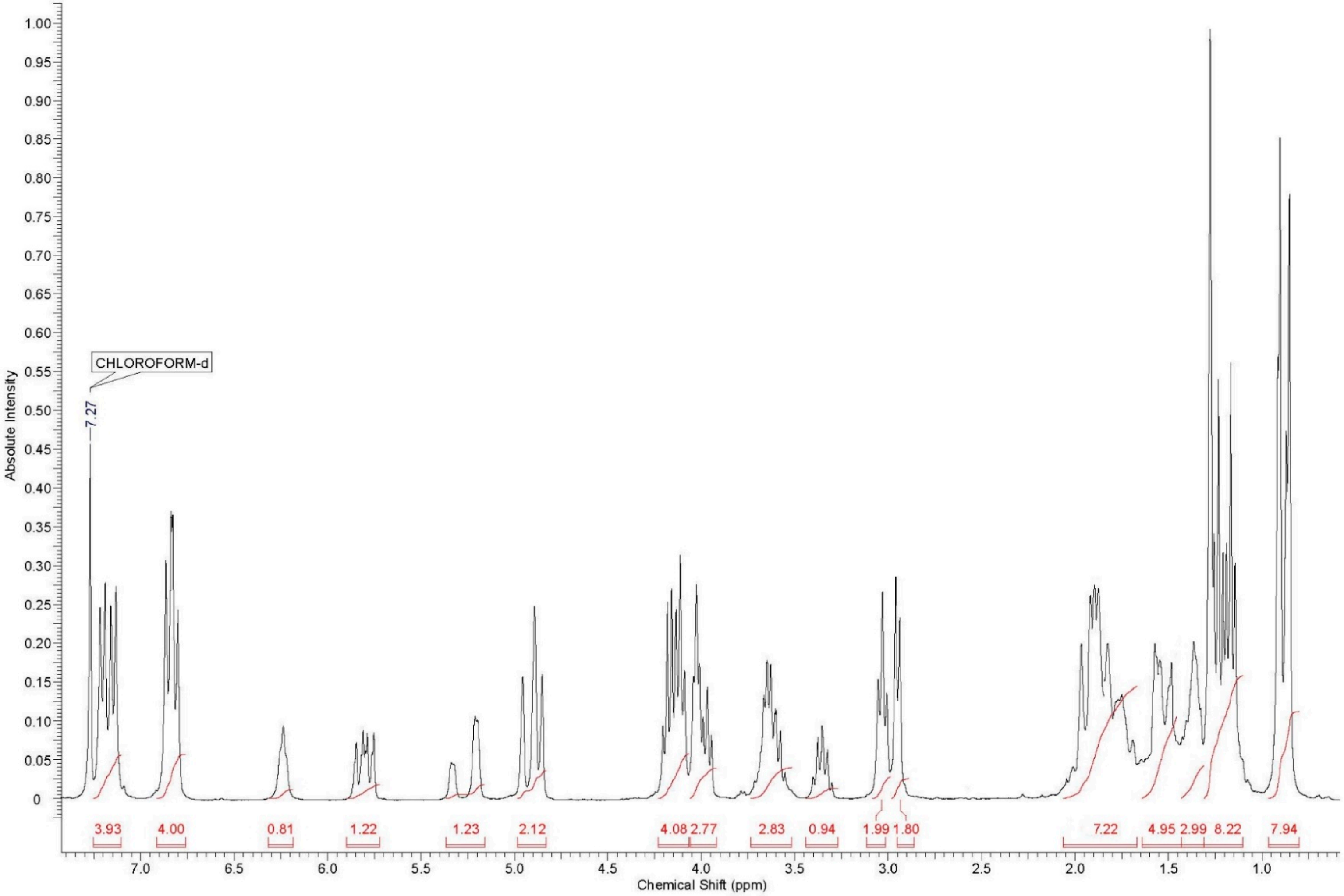

Figure S5. Ethyl (2S)-3-(4-{2-[4-(2-{[(1R,4aR,7S)-7-ethenyl-1,4a,7-trimethyl-1,2,3,4,4a,4b,5,6,7, 8,10,10a-dodecahydrophenanthren-1-yl]formamido}ethoxy)phenyl] ethoxy}phenyl)-2-ethoxypropanoate (**8a**) ^{1}H NMR spectrum

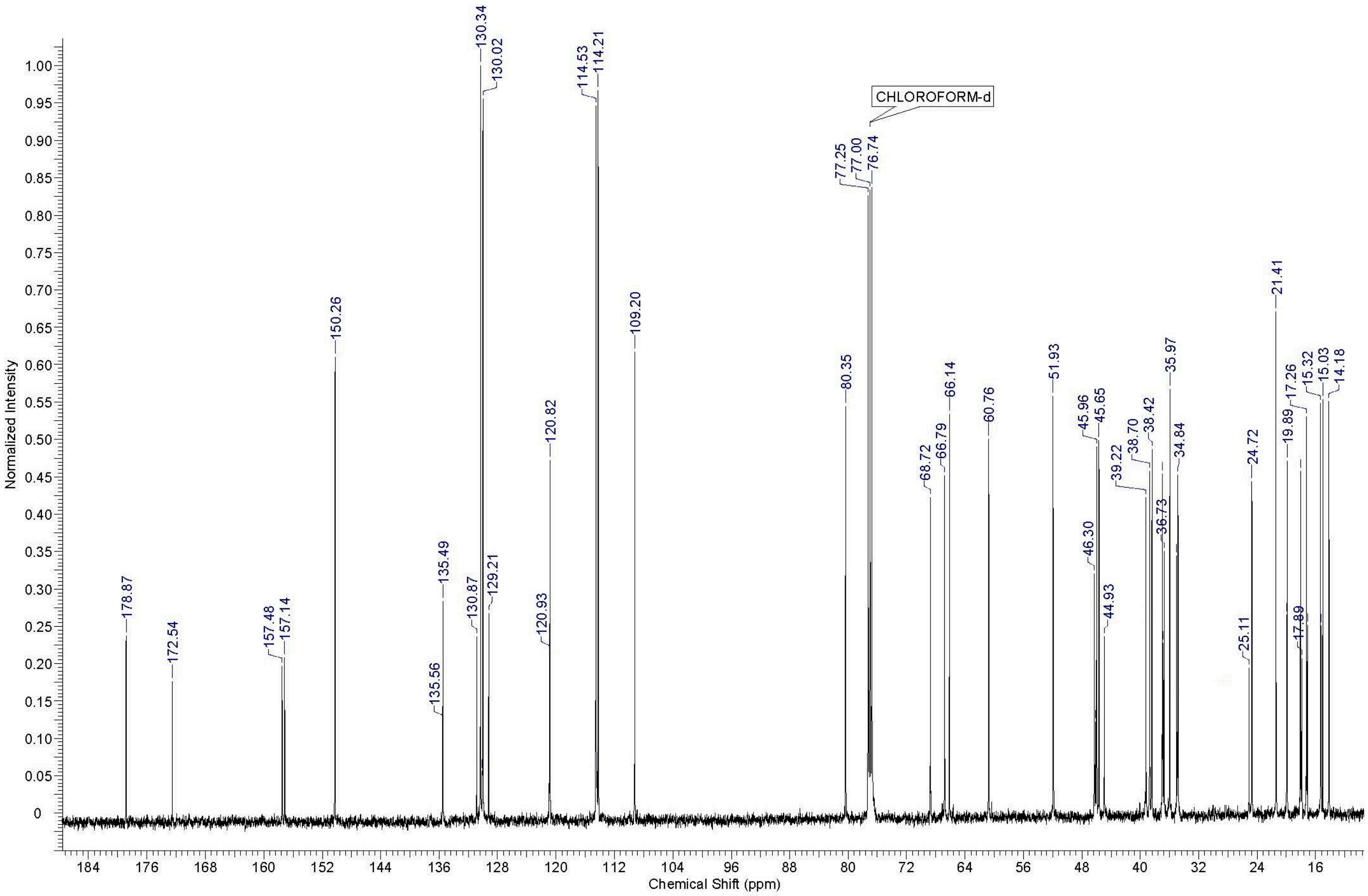

Figure S6. Ethyl (2S)-3-(4-{2-[4-(2-{[(1R,4aR,7S)-7-ethenyl-1,4a,7-trimethyl-1,2,3,4,4a,4b,5,6,7, 8,10,10a-dodecahydrophenanthren-1-yl]formamido}ethoxy)phenyl] ethoxy}phenyl)-2-ethoxypropanoate (**8a**) ^{13}C NMR spectrum

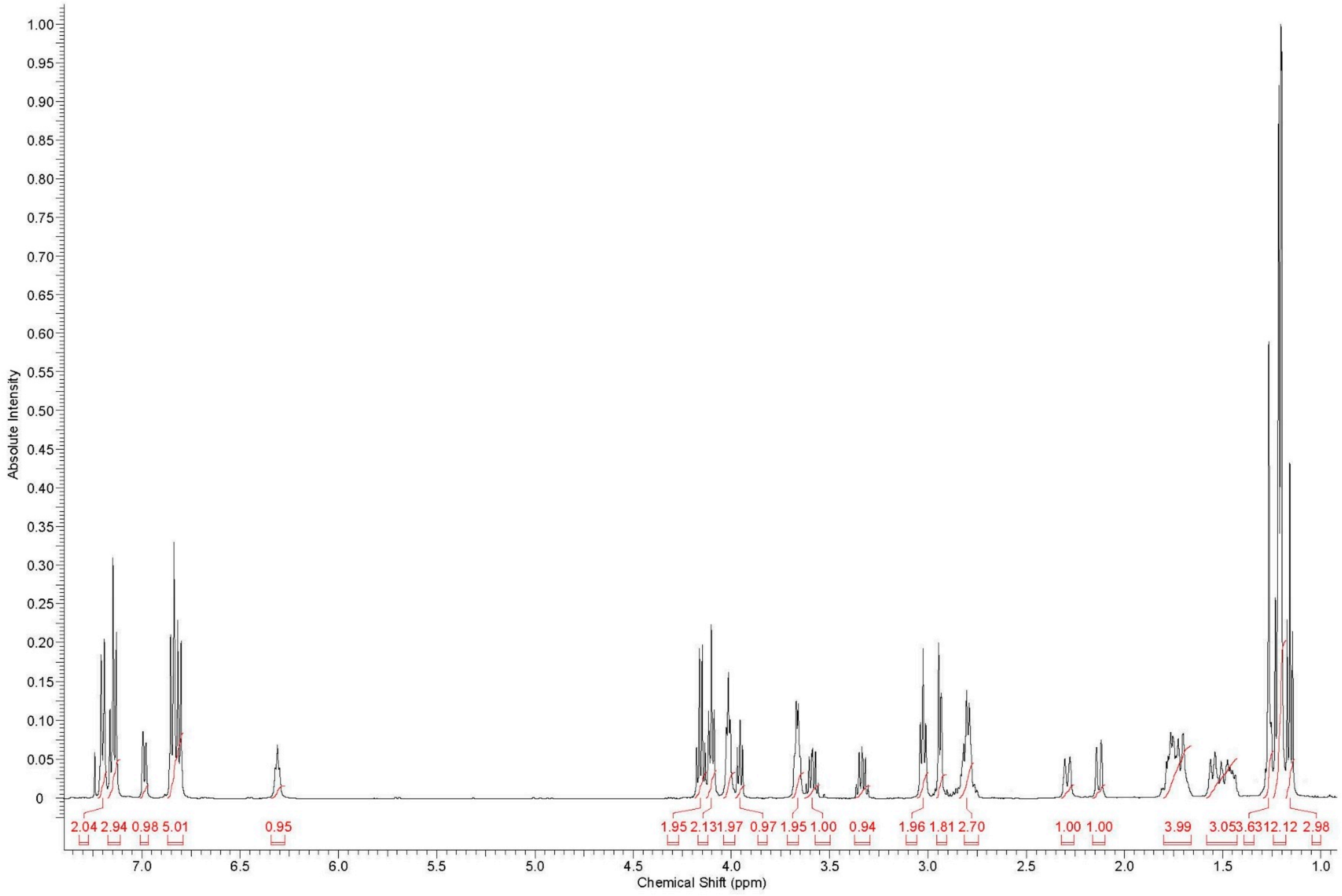

Figure S7. Ethyl (2S)-3-(4-{2-[4-(2-{[(1R,4aS,10aR)-1,4a-dimethyl-7-(propan-2-yl)-1,2,3,4,4a,9, 10,10a-octahydrophenanthren-1-yl]formamido}ethoxy)phenyl]ethoxy}phenyl)-2-ethoxypropanoate (**8b**) ^{1}H NMR spectrum

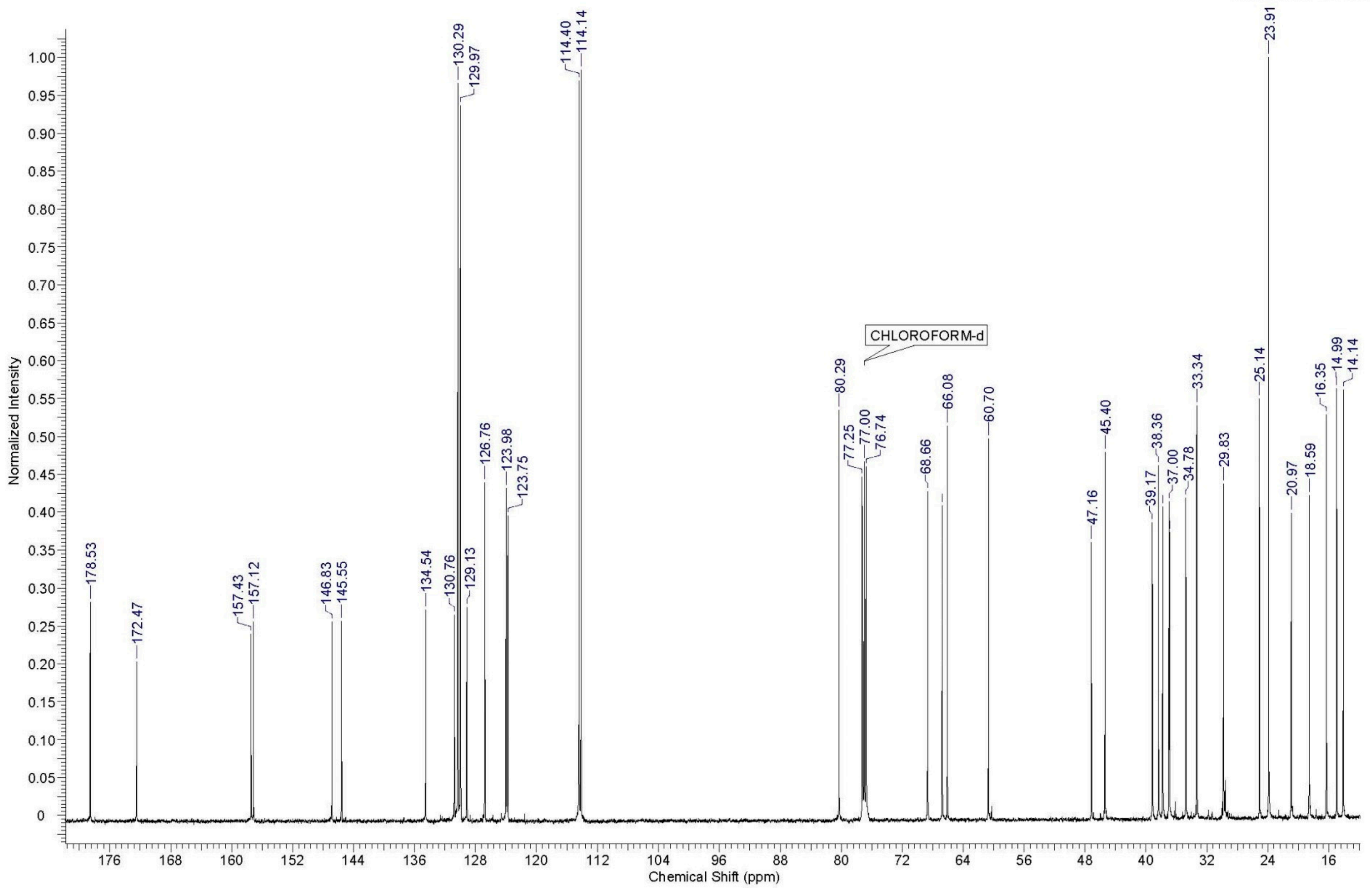

Figure S8. Ethyl (2S)-3-(4-{2-[4-(2-{[(1R,4aS,10aR)-1,4a-dimethyl-7-(propan-2-yl)-1,2,3,4,4a,9, 10,10a-octahydrophenanthren-1-yl]formamido}ethoxy)phenyl]ethoxy}phenyl)-2-ethoxypropanoate (**8b**) ^{13}C NMR spectrum

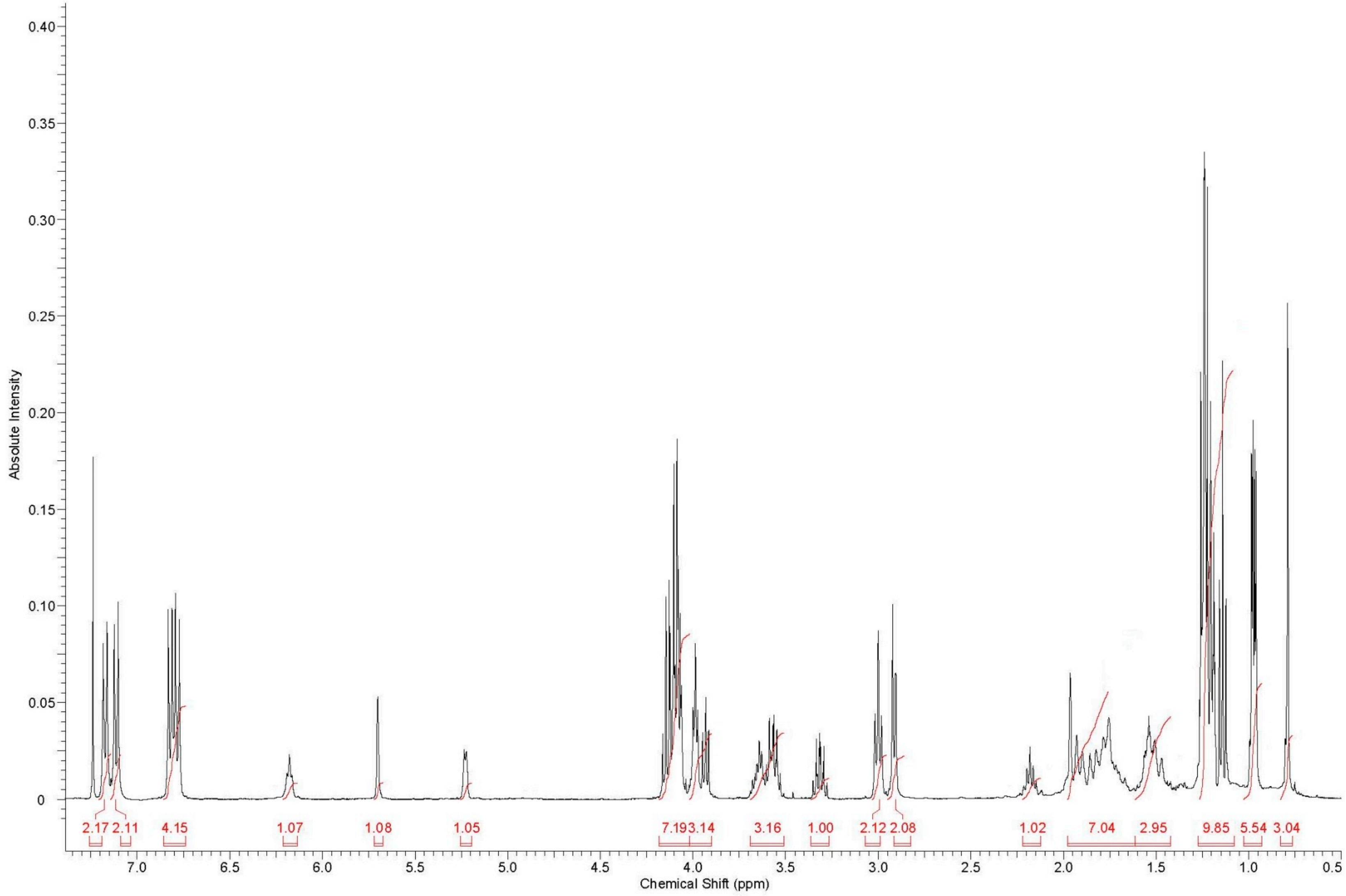

Figure S9. Ethyl (2S)-3-(4-{2-[4-(2-{[(1R,4aR,10aR)-1,4a-dimethyl-7-(propan-2-yl)-1,2,3,4,4a,4b,5, 6,10,10a-decahydrophenanthren-1-yl]formamido}ethoxy)phenyl]ethoxy}phenyl)-2-ethoxypropanoate (**8c**) ^{1}H NMR spectrum

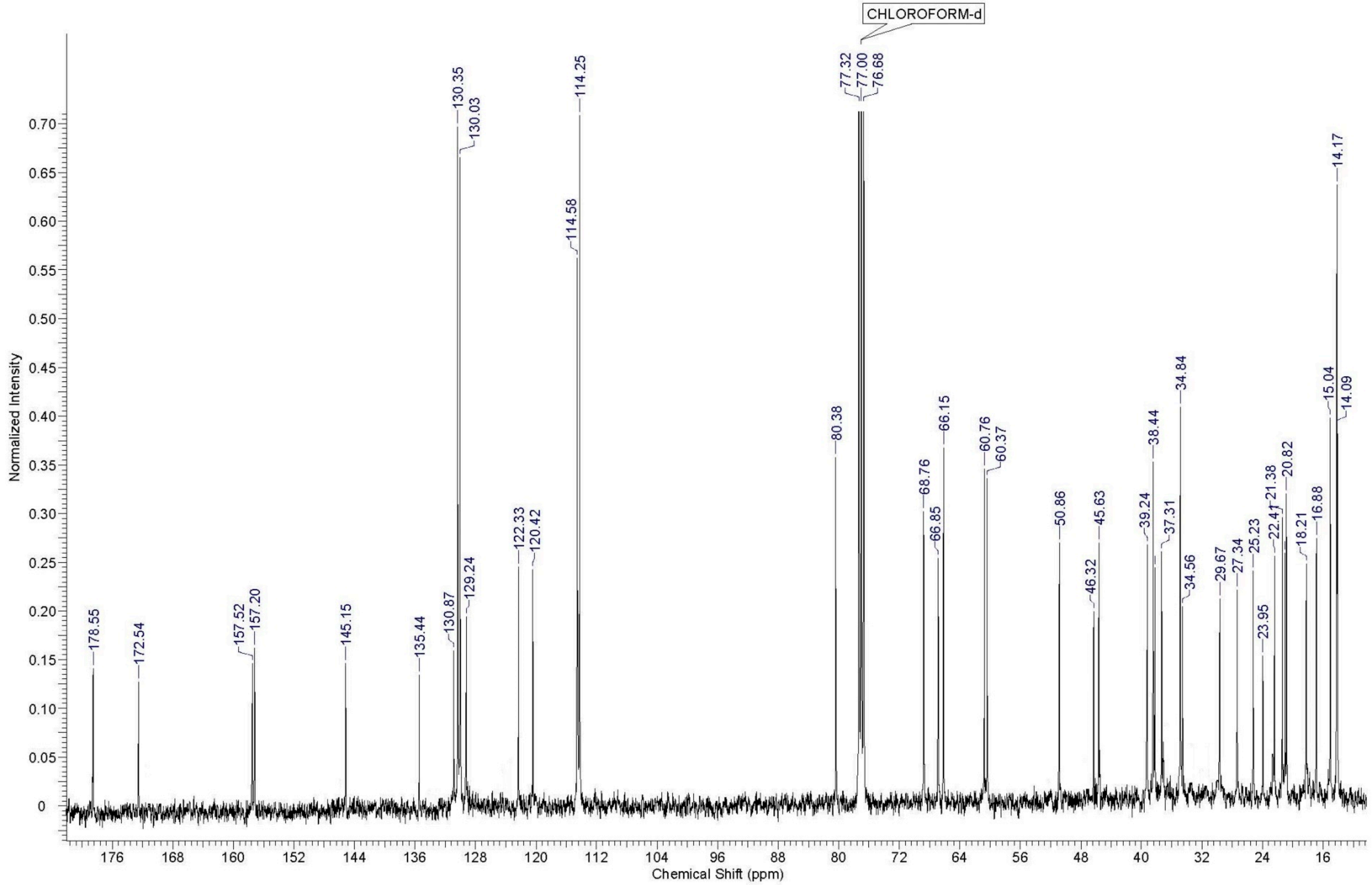

Figure S10. Ethyl (2S)-3-(4-{2-[4-(2-{[(1R,4aR,10aR)-1,4a-dimethyl-7-(propan-2-yl)-1,2,3,4,4a,4b,5, 6,10,10a-decahydrophenanthren-1-yl]formamido}ethoxy)phenyl]ethoxy}phenyl)-2-ethoxypropanoate (**8c**) ^{13}C NMR spectrum

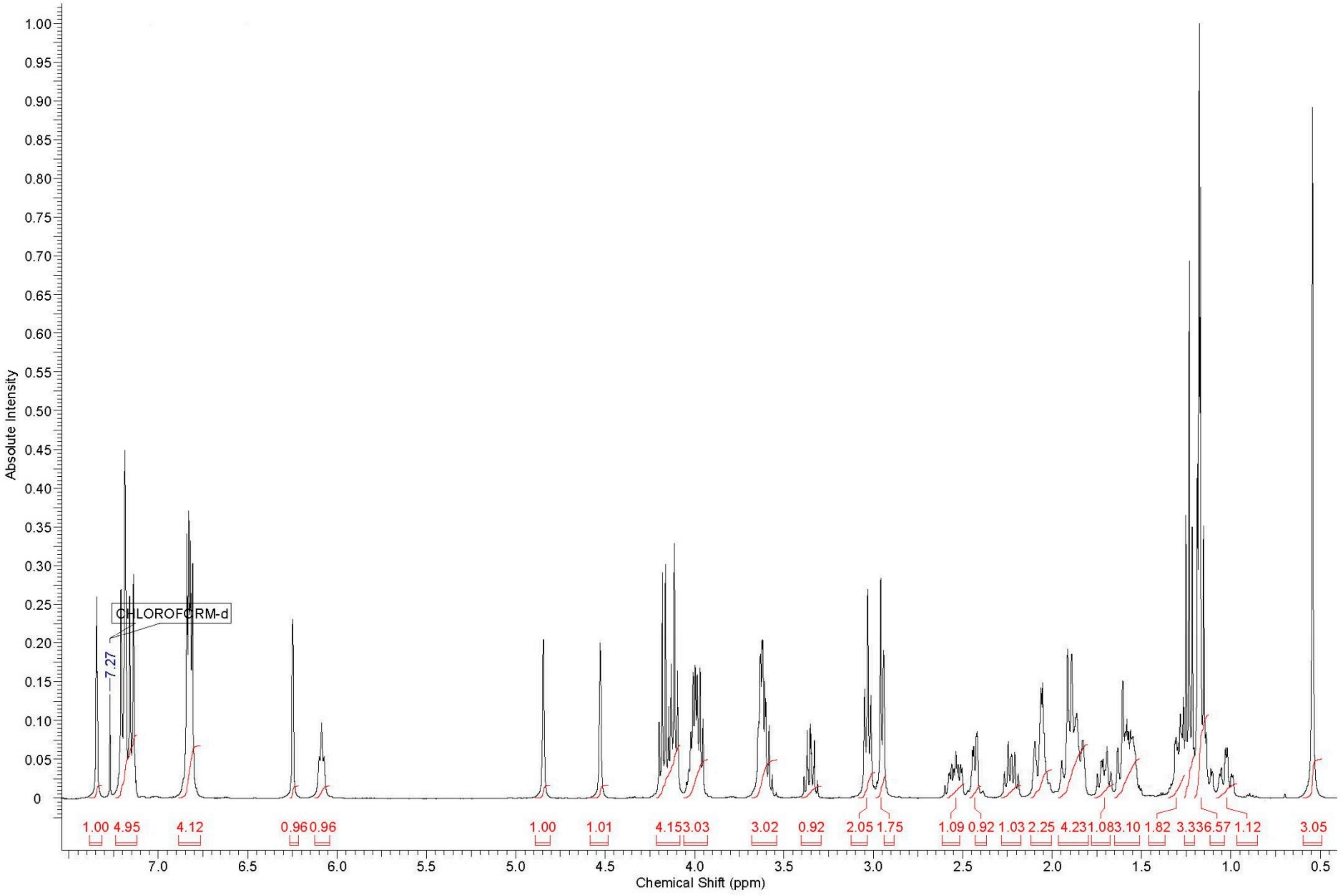

Figure S11. Ethyl (2S)-3-(4-{2-[4-(2-{[(1R,4aR,5S)-5-[2-(furan-3-yl)ethyl]-1,4a-dimethyl-6-methyl idenedecahydronaphthalen-1-yl]formamido}ethoxy)phenyl]ethoxy}phenyl)-2-ethoxypropanoate (**8d**) ^{1}H NMR spectrum

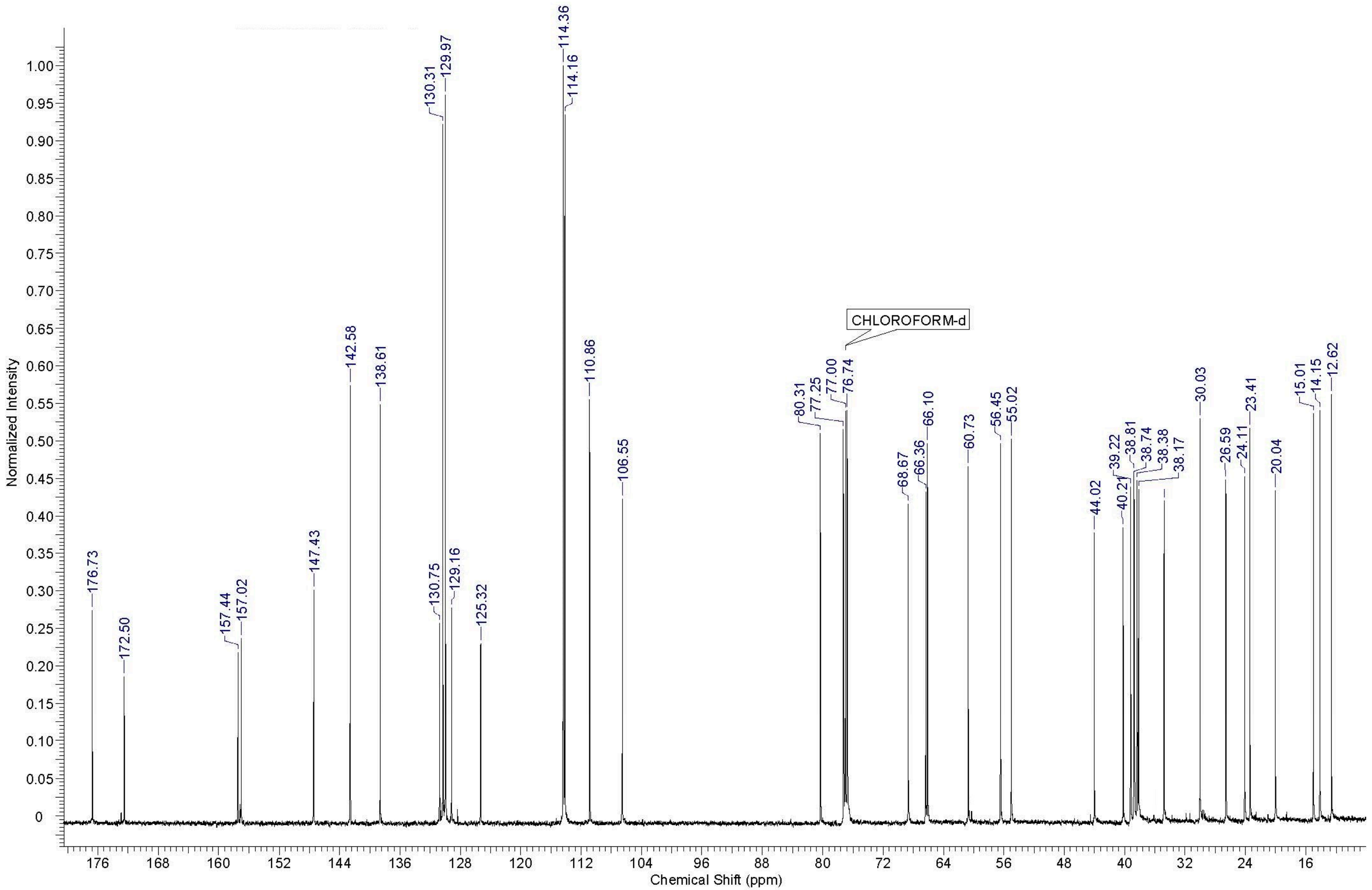

Figure S12. Ethyl (2S)-3-(4-{2-[4-(2-{[(1R,4aR,5S)-5-[2-(furan-3-yl)ethyl]-1,4a-dimethyl-6-methyl idenedecahydronaphthalen-1-yl]formamido}ethoxy)phenyl]ethoxy}phenyl)-2-ethoxypropanoate **(8d)** ^{13}C NMR spectrum

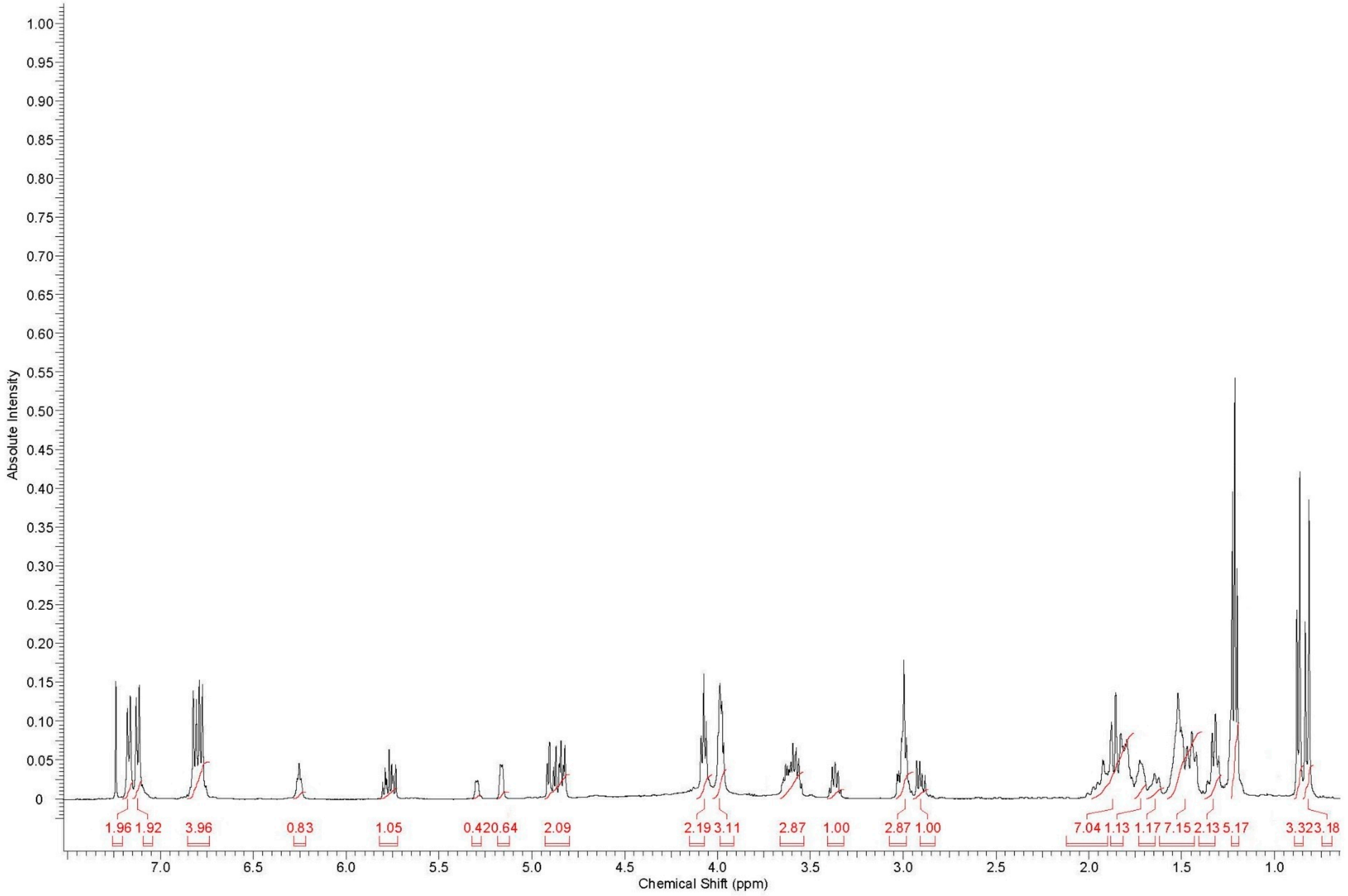

Figure S13. (2S)-3-(4-{2-[4-(2-{[(1R,4aR,7S)-7-ethenyl-1,4a,7-trimethyl-1,2,3,4,4a,4b,5,6,7,8,10, 10a-dodecahydrophenanthren-1-yl]formamido}ethoxy)phenyl]ethoxy}phenyl)-2-ethoxy-propanoic acid (**9a)** ^{1}H NMR spectrum

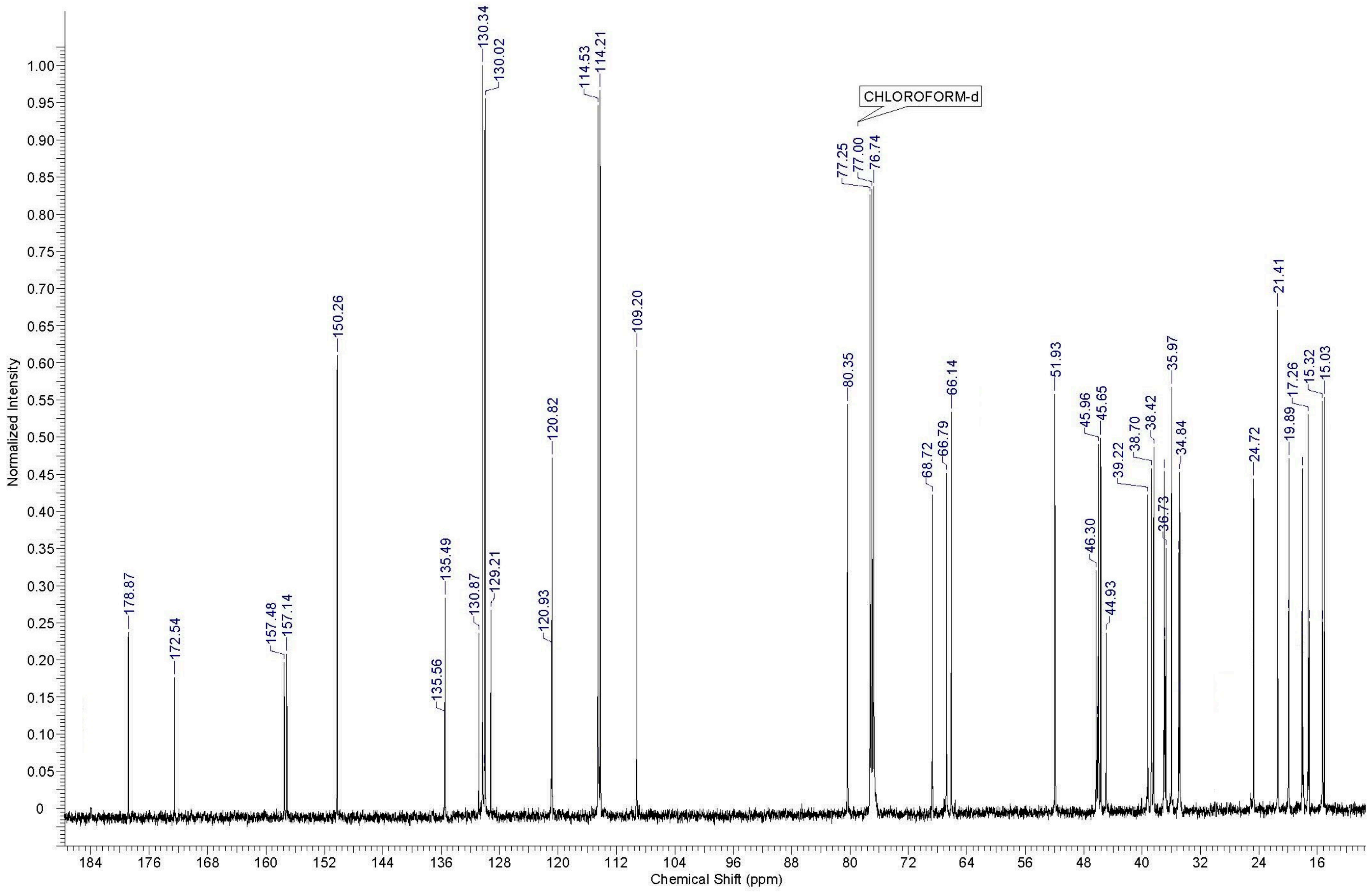

Figure S14. (2S)-3-(4-{2-[4-(2-{[(1R,4aR,7S)-7-ethenyl-1,4a,7-trimethyl-1,2,3,4,4a,4b,5,6,7,8,10, 10a-dodecahydrophenanthren-1-yl]formamido}ethoxy)phenyl]ethoxy}phenyl)-2-ethoxy-propanoic acid (**9a**) ^{13}C NMR spectrum

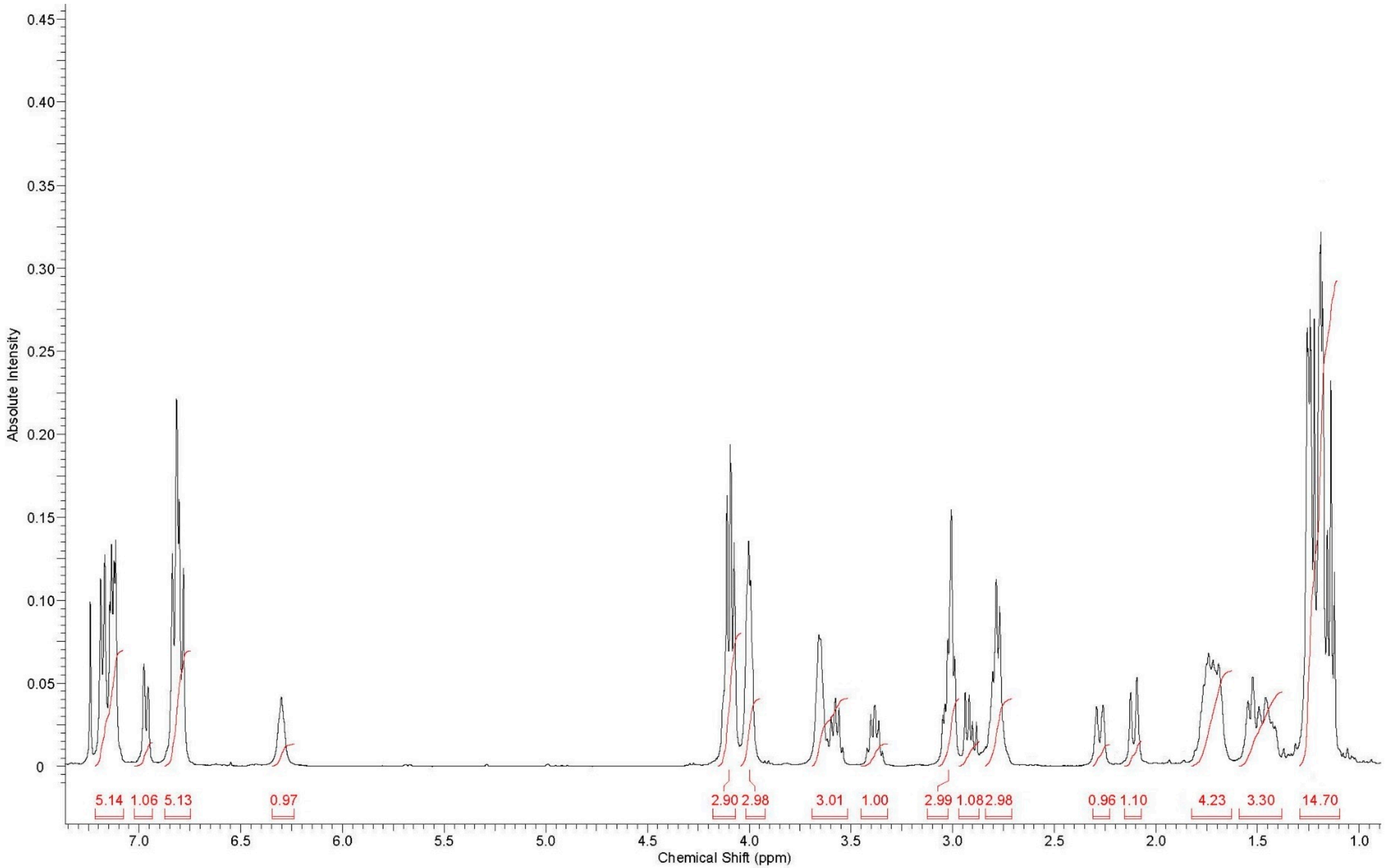

Figure S15. (2S)-3-(4-{2-[4-(2-{[(1R,4aS,10aR)-1,4a-dimethyl-7-(propan-2-yl)-1,2,3,4,4a,9,10,10a-octahydrophenanthren-1-yl]formamido}ethoxy)phenyl]ethoxy}phenyl)-2-ethoxypropanoic acid (**9b**) ^{1}H NMR spectrum

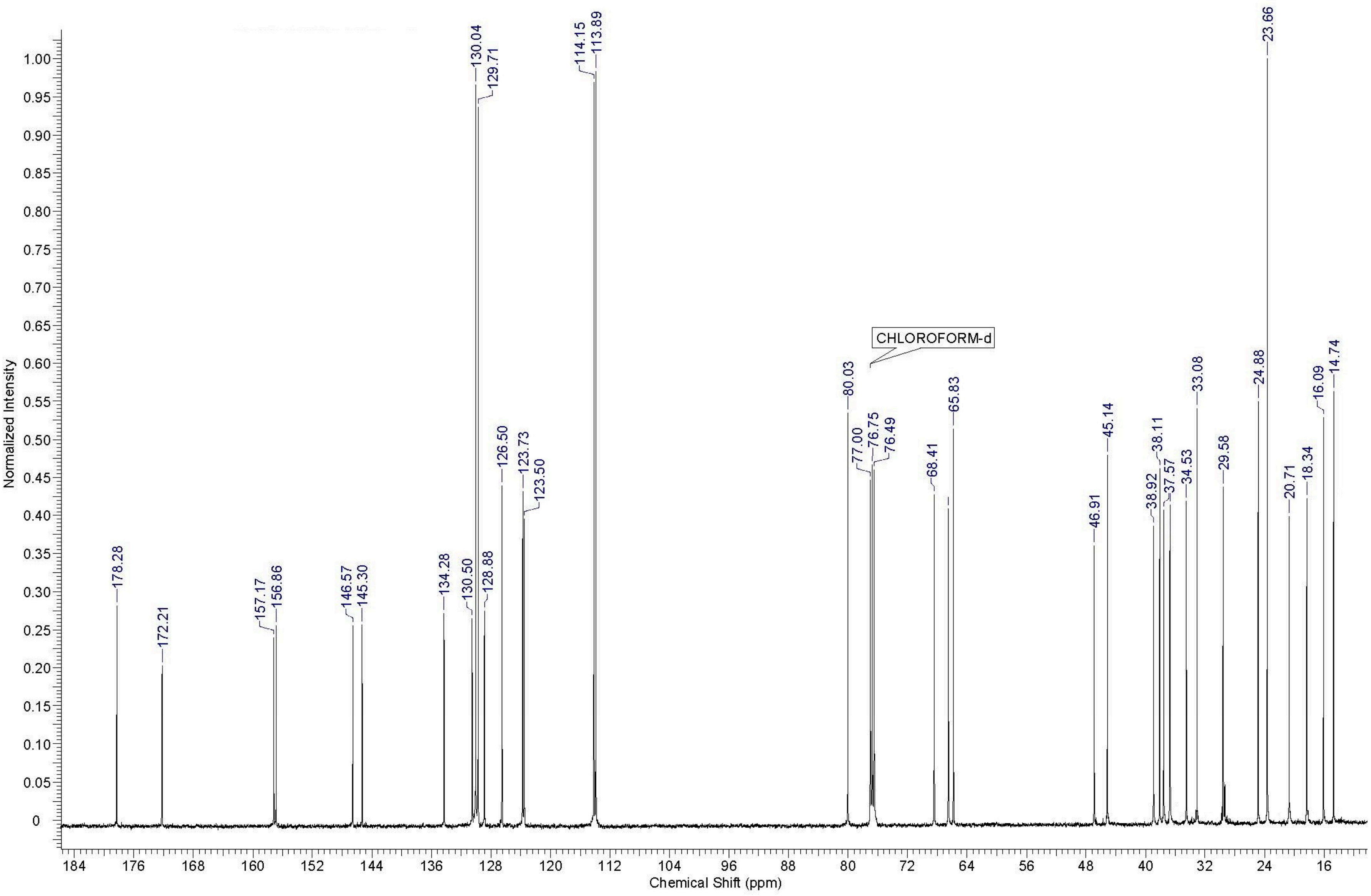

Figure S16. (2S)-3-(4-{2-[4-(2-{[(1R,4aS,10aR)-1,4a-dimethyl-7-(propan-2-yl)-1,2,3,4,4a,9,10,10a-octahydrophenanthren-1-yl]formamido}ethoxy)phenyl]ethoxy}phenyl)-2-ethoxypropanoic acid (**9b)** ^{13}C NMR spectrum

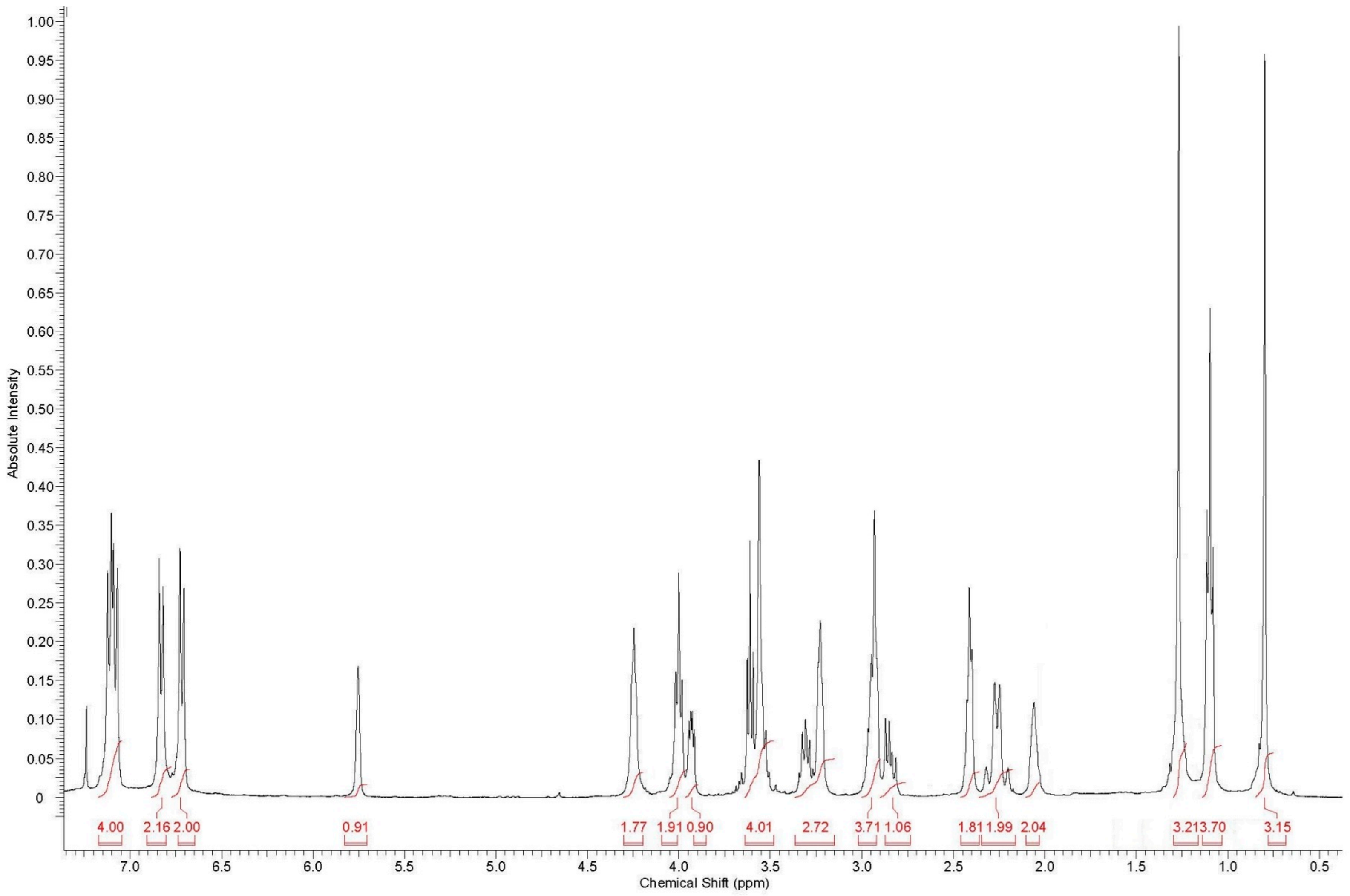

Figure S17. {2-[4-(2-{4-[(2S)-2-carboxy-2-ethoxyethyl]phenoxy}ethyl)phenoxy]ethyl}({[(1R,5S)-6,6-dimethylbicyclo[3.1.1]hept-2-en-2-yl]methyl})azanium chloride (**7a**) ^{1}H NMR spectrum

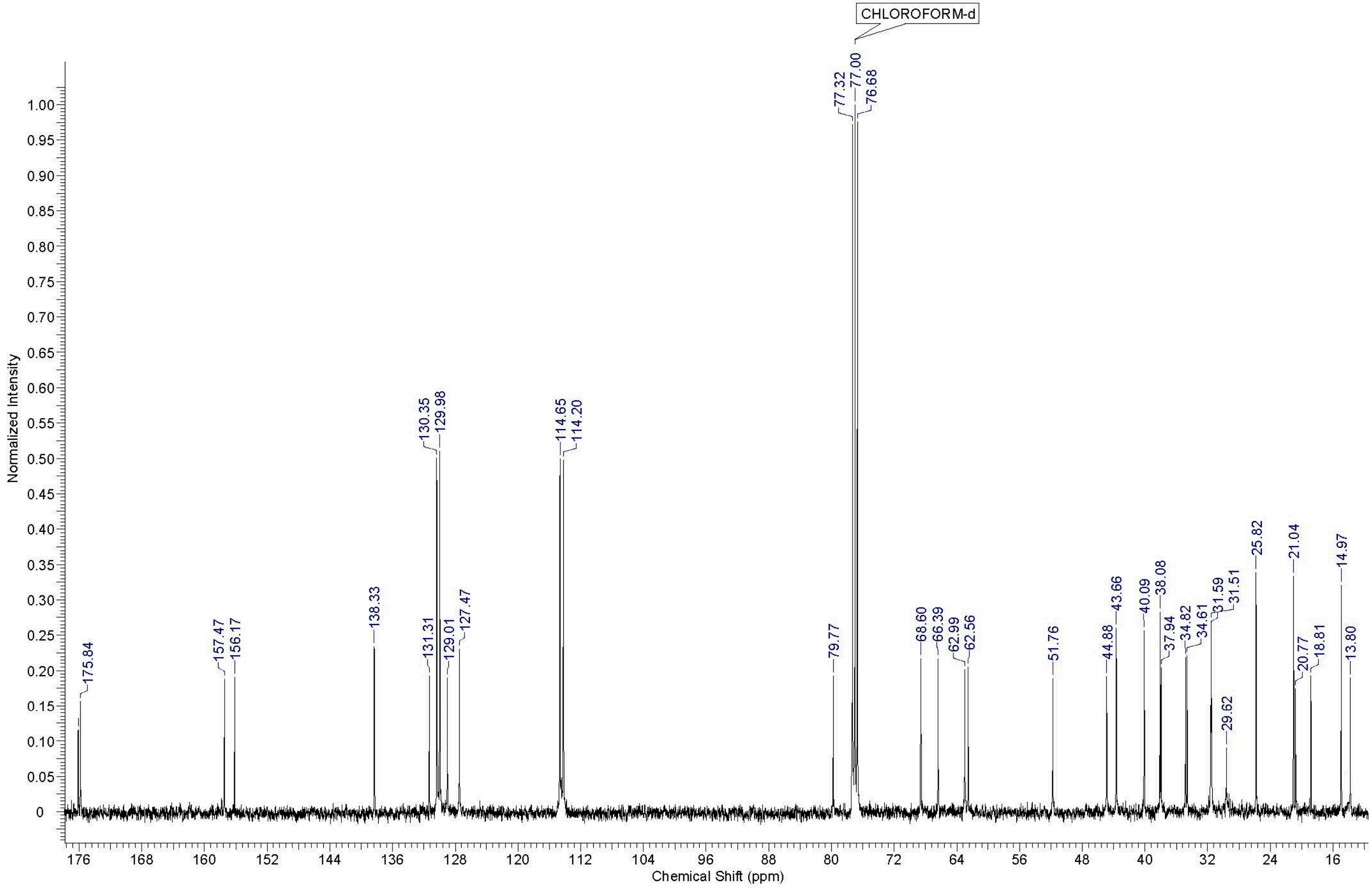

Figure S18. {2-[4-(2-{4-[(2S)-2-carboxy-2-ethoxyethyl]phenoxy}ethyl)phenoxy]ethyl}({[(1R,5S)-6,6-dimethylbicyclo[3.1.1]hept-2-en-2-yl]methyl})azanium chloride (**7a**) ^{13}C NMR spectrum

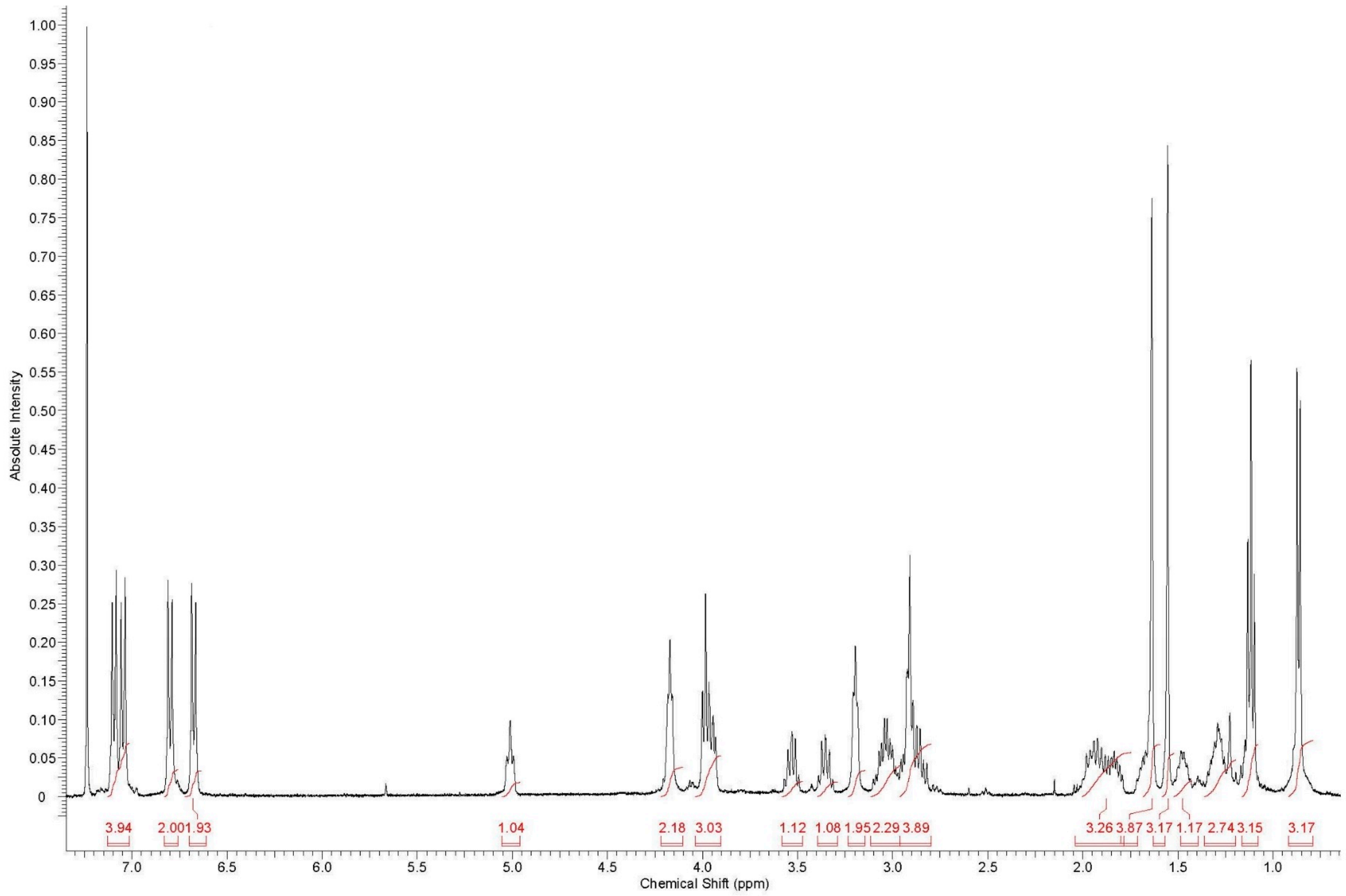

Figure S19. {2-[4-(2-{4-[(2S)-2-carboxy-2-ethoxyethyl]phenoxy}ethyl)phenoxy]ethyl}(3,7-dimethyloct-6-en-1-yl)azanium chloride (**7b**) ^{1}H NMR spectrum

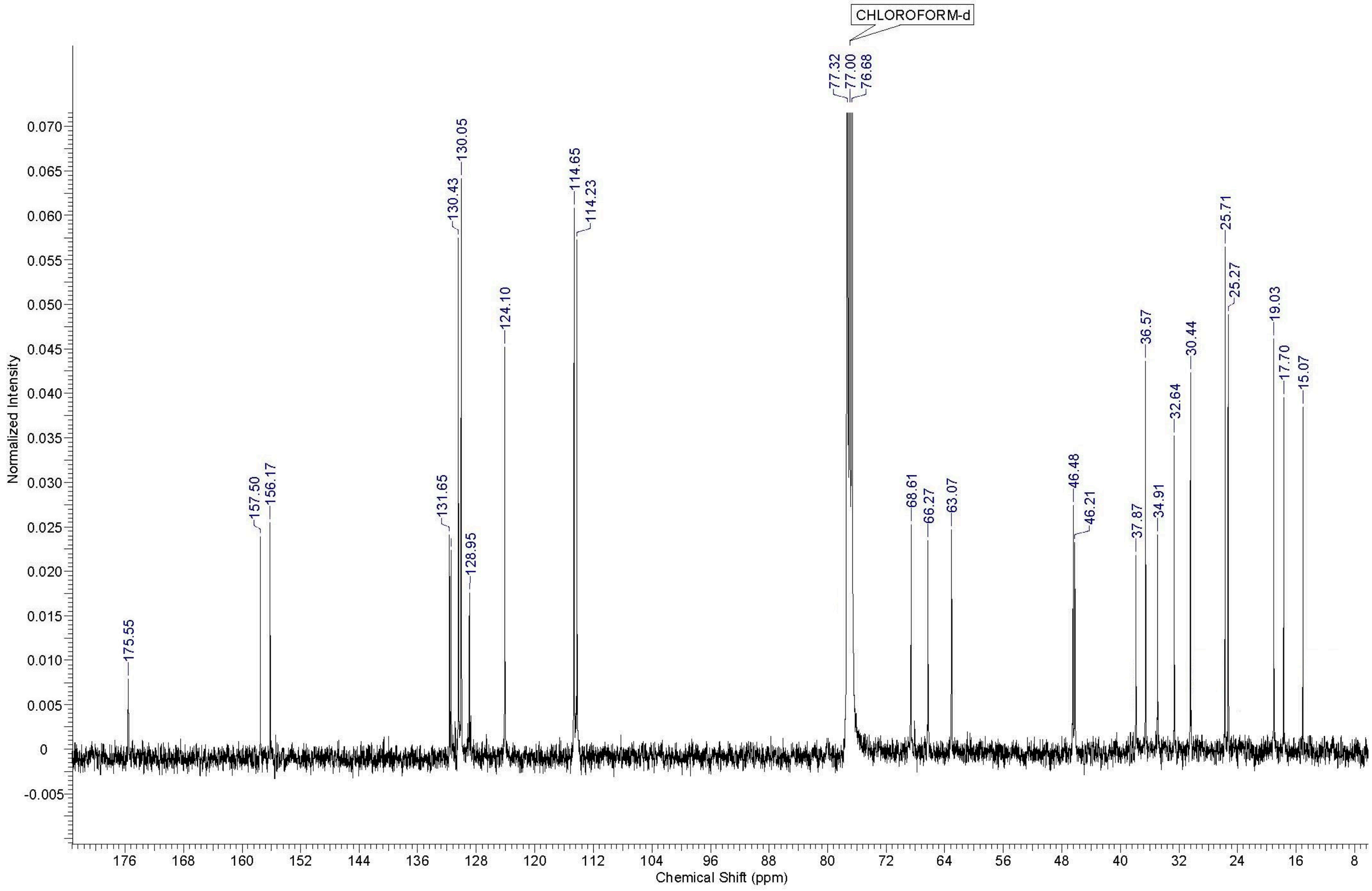

Figure S20. {2-[4-(2-{4-[(2S)-2-carboxy-2-ethoxyethyl]phenoxy}ethyl)phenoxy]ethyl}(3,7-dimethyloct-6-en-1-yl)azanium chloride (**7b**) ^{13}C NMR spectrum

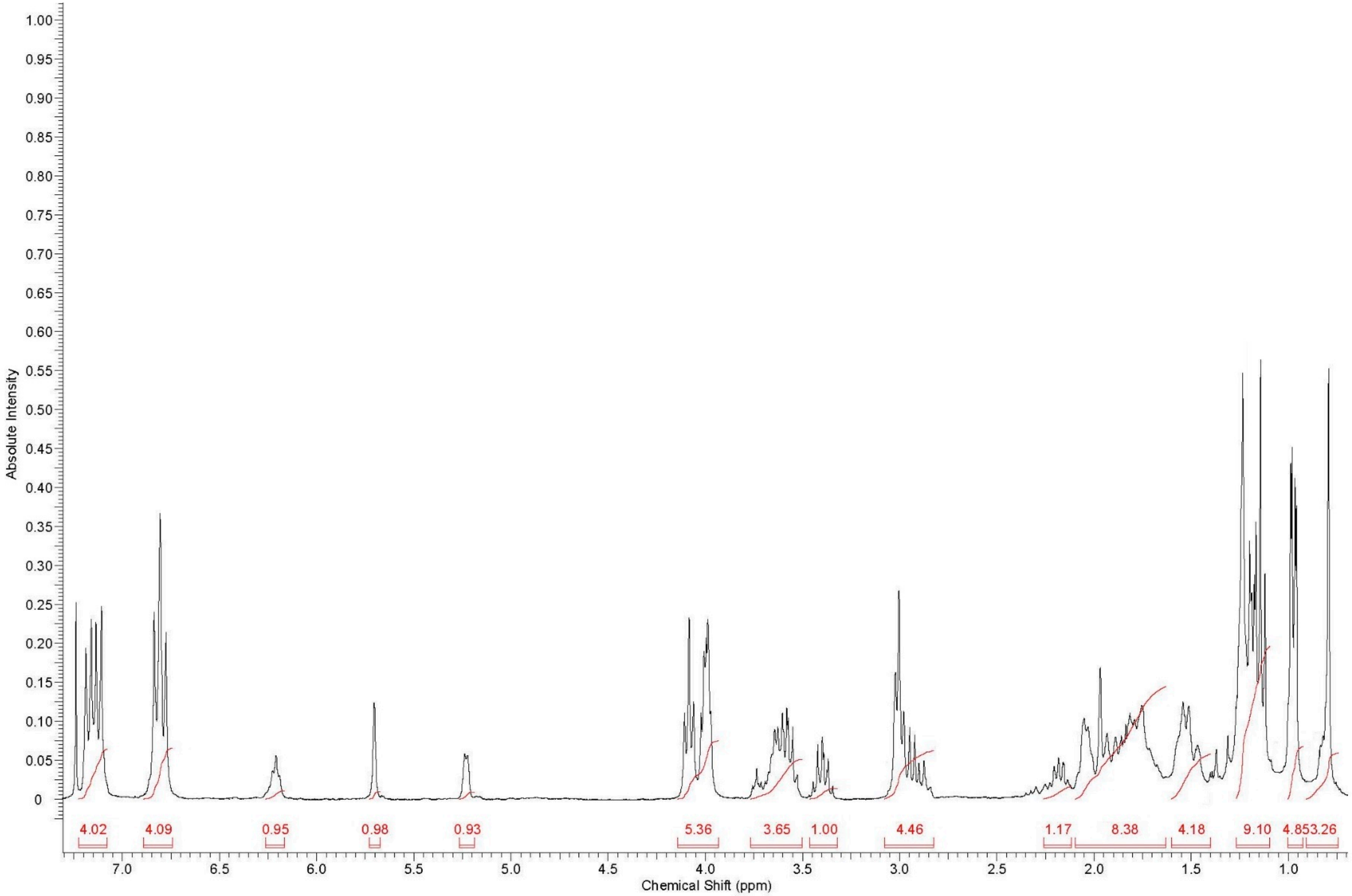

Figure S21. (2S)-3-(4-{2-[4-(2-{[(1R,4aR,10aR)-1,4a-dimethyl-7-(propan-2-yl)-1,2,3,4,4a,4b,5,6,10,10a-decahydrophenanthren-1-yl]formamido}ethoxy)phenyl]ethoxy}phenyl)-2-ethoxypropanoic acid (**9c**) ^{1}H NMR spectrum

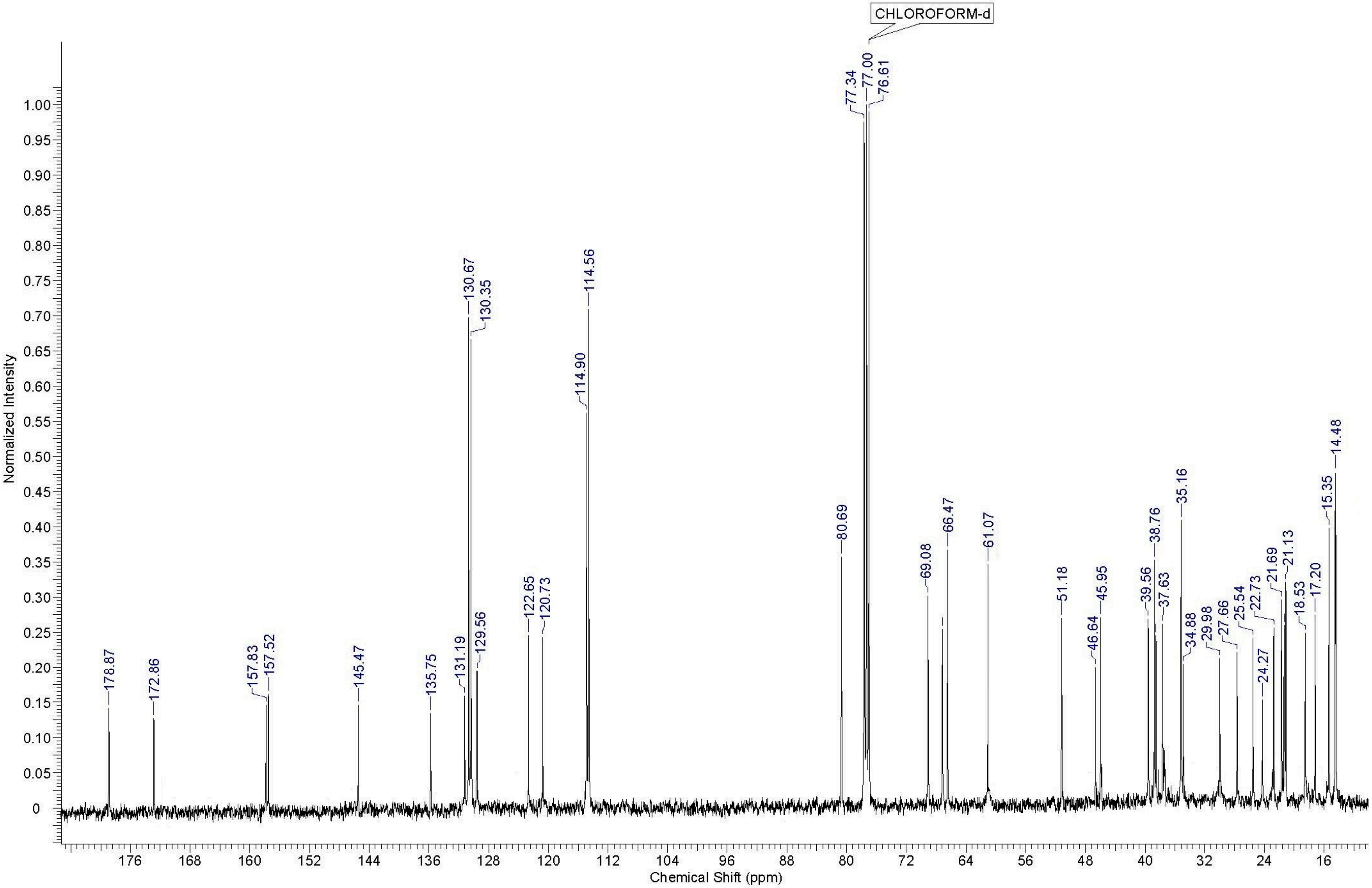

Figure S22. (2S)-3-(4-{2-[4-(2-{[(1R,4aR,10aR)-1,4a-dimethyl-7-(propan-2-yl)-1,2,3,4,4a,4b,5,6,10,10a-decahydrophenanthren-1-yl]formamido}ethoxy)phenyl]ethoxy}phenyl)-2-ethoxypropanoic acid (**9c**) ^{13}C NMR spectrum

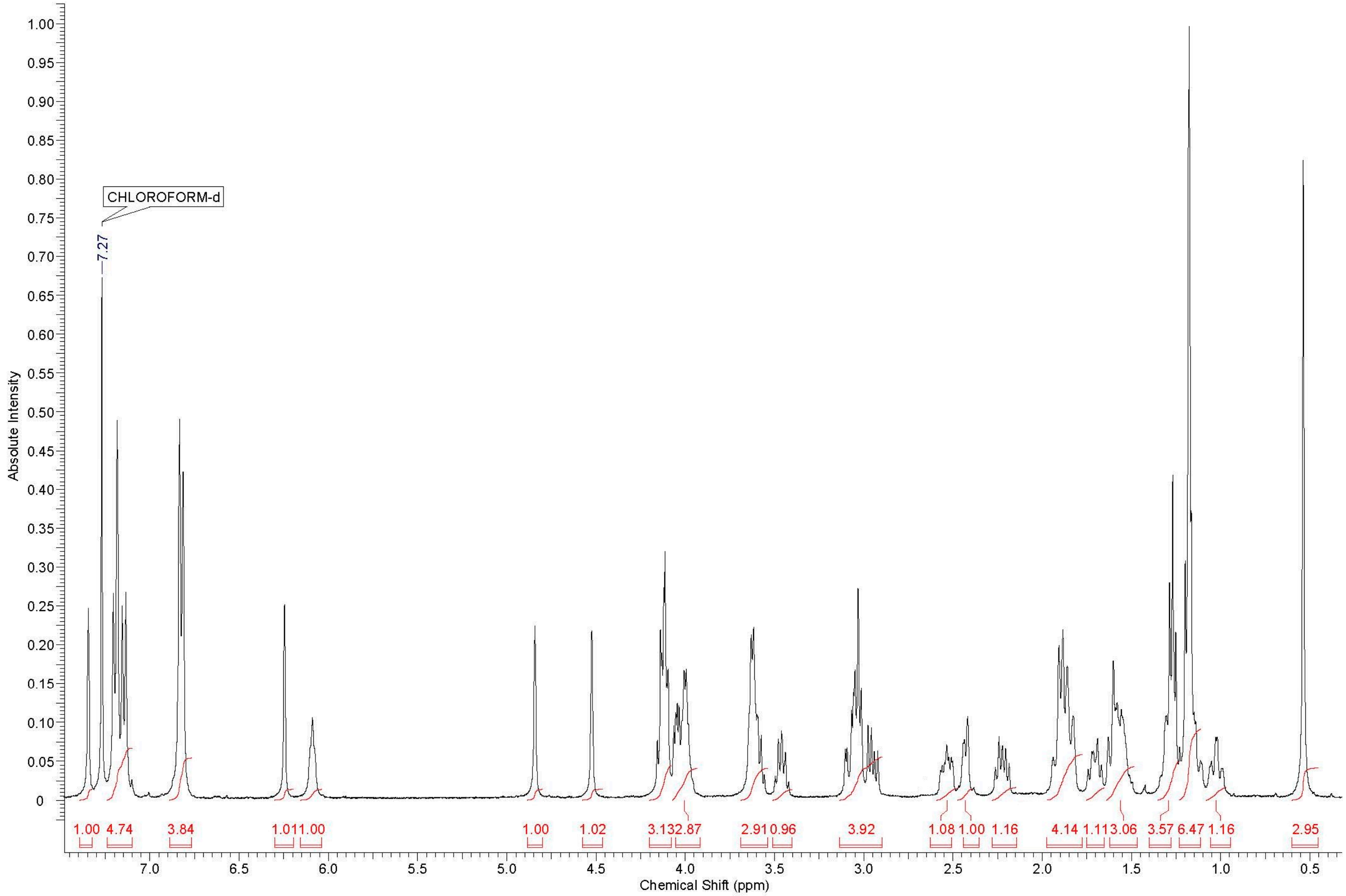

Figure S23. (2S)-3-(4-{2-[4-(2-{[(1R,4aR,5S)-5-[2-(furan-3-yl)ethyl]-1,4a-dimethyl-6-methylidene-decahydronaphthalen-1-yl]formamido}ethoxy)phenyl]ethoxy}phenyl)-2-ethoxypropanoic acid (**9d**) ^{1}H NMR spectrum

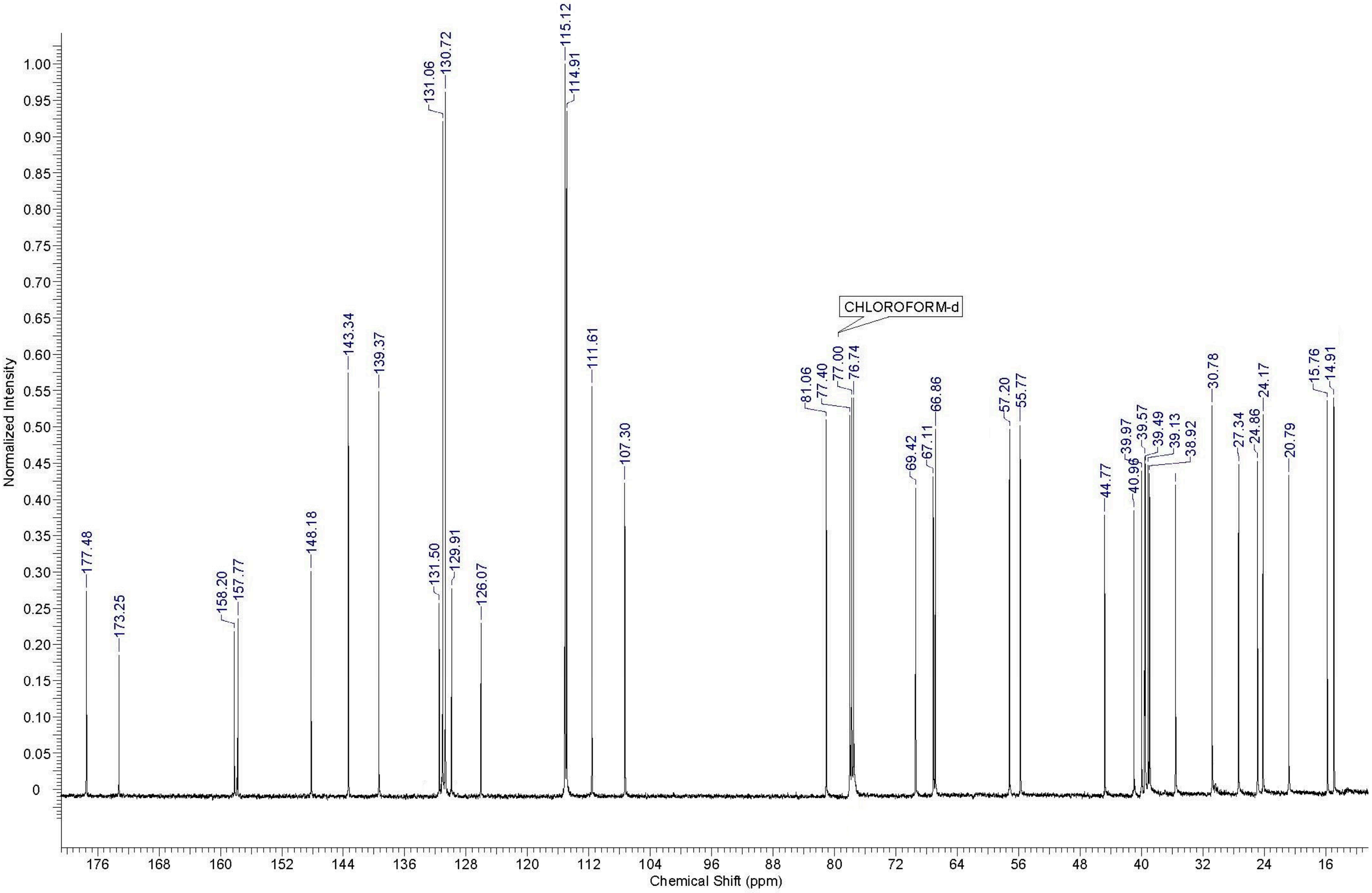

Figure S24. (2S)-3-(4-{2-[4-(2-{[(1R,4aR,5S)-5-[2-(furan-3-yl)ethyl]-1,4a-dimethyl-6-methylidene-decahydronaphthalen-1-yl]formamido}ethoxy)phenyl]ethoxy}phenyl)-2-ethoxypropanoic acid (**9d**) ^{13}C NMR spectrum
